# Long-read based detection of large copy number variants with potential functional significance using the ContextSV structural variant caller

**DOI:** 10.1093/nargab/lqag108

**Published:** 2026-09-09

**Authors:** Jonathan Elliot Perdomo, Mian Umair Ahsan, Jasmine Akoto, James Bauer, Naiara Akizu, Kai Wang

**Affiliations:** Raymond G. Perelman Center for Cellular and Molecular Therapeutics, Children’s Hospital of Philadelphia, Philadelphia, PA 19104, United States; School of Biomedical Engineering and Science, Drexel University, Philadelphia, PA 19104, United States; Raymond G. Perelman Center for Cellular and Molecular Therapeutics, Children’s Hospital of Philadelphia, Philadelphia, PA 19104, United States; Raymond G. Perelman Center for Cellular and Molecular Therapeutics, Children’s Hospital of Philadelphia, Philadelphia, PA 19104, United States; Raymond G. Perelman Center for Cellular and Molecular Therapeutics, Children’s Hospital of Philadelphia, Philadelphia, PA 19104, United States; Raymond G. Perelman Center for Cellular and Molecular Therapeutics, Children’s Hospital of Philadelphia, Philadelphia, PA 19104, United States; Department of Pathology and Laboratory Medicine, University of Pennsylvania, Philadelphia, PA 19104, United States; Raymond G. Perelman Center for Cellular and Molecular Therapeutics, Children’s Hospital of Philadelphia, Philadelphia, PA 19104, United States; Department of Pathology and Laboratory Medicine, University of Pennsylvania, Philadelphia, PA 19104, United States

## Abstract

Long-read sequencing enables improved detection of structural variants (SVs) in the human genome due to its substantially increased read lengths. However, currently widely used long-read SV callers primarily rely on alignment-based evidence, limiting their ability to detect large and complex SVs and potentially missing disease-relevant events. To address these limitations, we developed ContextSV, a framework that integrates alignment evidence with copy number predictions derived from sequencing coverage and single-nucleotide variant allele frequencies to improve SV detection, particularly for large copy number variants (CNVs). We additionally developed ContextScore, a machine learning–based classification model to assign SV confidence scores based on genomic context features and integrated it within ContextSV. Through benchmarking analyses on both simulated and real datasets, we demonstrate that ContextSV improves detection of large CNVs and inversions that may be missed by existing long-read SV callers. We further illustrate its utility by identifying and experimentally validating multiple large SVs in the KOLF2.1J reference stem cell line that were not detected by other methods. Collectively, our results demonstrate that ContextSV serves as a valuable complement to existing long-read SV detection approaches by improving sensitivity for large and clinically relevant SVs.

## Graphical Abstract

**Graphical Abstract:**
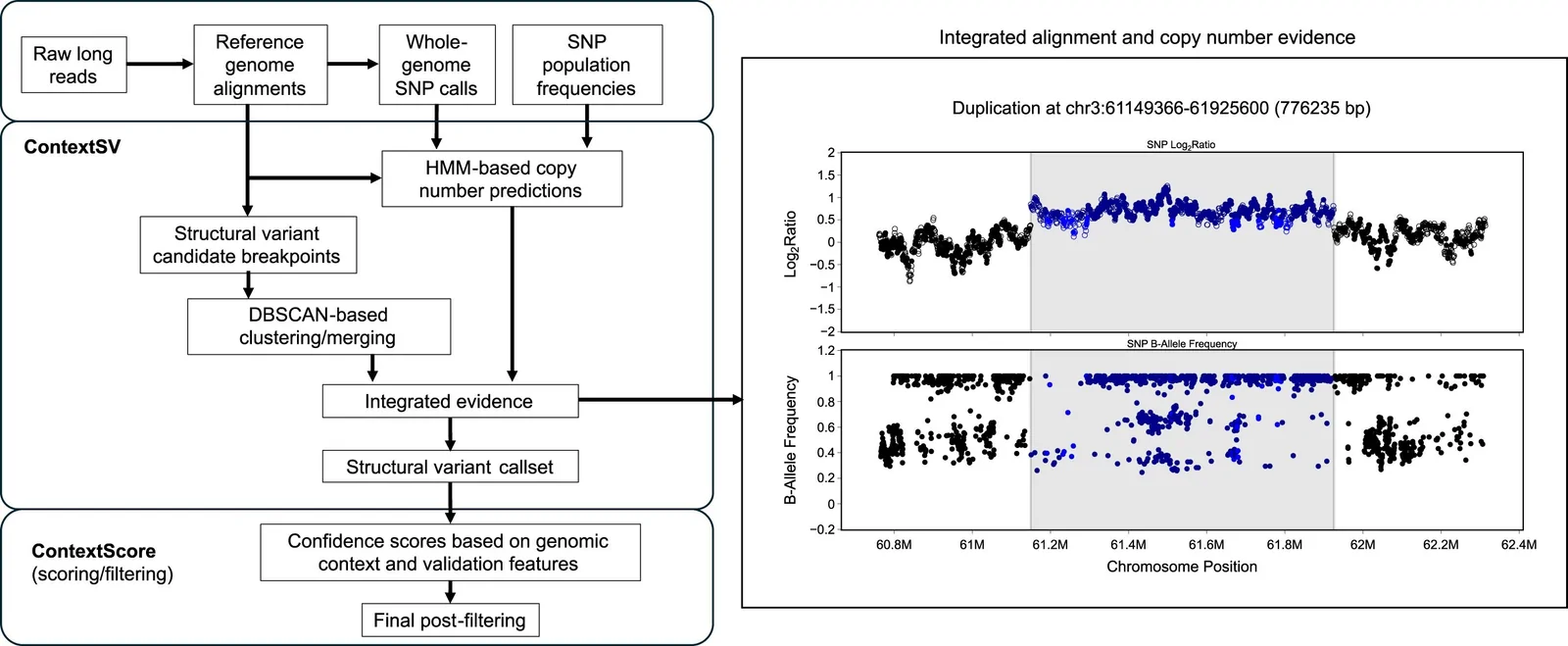

## Introduction

Characterizing human genomic variants between individuals and populations is crucial to our understanding of phenotypic variation and disease susceptibility. Structural variants (SVs) are the largest source of genomic differences, and range in size from ∼50 base pairs (bp) to several megabases [1]. SVs can involve sequence insertions, deletions, duplications, inversions, and/or inter-/intra-chromosomal translocations, and are generally defined relative to a linear reference genome (e.g. GRCh37 and GRCh38) or to a graph-based pangenome reference.

Advancements in whole-genome long-read sequencing technologies, such as Pacific Biosciences (PacBio) and Oxford Nanopore Technologies (ONT), enable the detection of larger and more complex SVs relative to short-read approaches due to increased read lengths (∼10–30 kilobases) while maintaining a high accuracy [2]. However, existing long-read SV detection methods have limitations in the size and complexity of detectable SVs, primarily due to reliance on alignment evidence for SV calling and filtering.

Long-read SV calling algorithms fall into two major categories: alignment- and assembly-based methods [3]. Alignment-based methods identify SVs directly from read alignments to a linear reference genome, while assembly-based methods require assembling reads into contigs prior to alignment. Although assembly-based methods can identify larger SVs, they require high coverage, and read assembly requires significant computational resources. Therefore, alignment-based methods are generally preferred for most applications, as they can obtain high-quality SV callsets within a reasonable computational time and/or with limited long-read coverage.

Current widely used alignment-based long-read SV callers such as Sniffles2 [4], cuteSV [5], and Delly [6] utilize similar approaches. Insertions, deletions, split alignments, and clipped bases from alignment records are used to identify and classify SVs. Split alignments occur when more than one potential read alignment is identified by the aligner. They provide important evidence for large events exceeding read lengths as well as for inversions and translocations. SVs are classified based on distance and orientation between split alignments on the read versus on the aligned reference. Clipped bases also provide supplementary evidence for breakpoints in larger SVs. Genotypes are predicted based on read support for reference versus alternate alleles. The major challenges in identifying SVs from alignment records alone include precise merging of breakpoint evidence, and precise filtering of false positives, largely due to alignment ambiguities in highly repetitive or unresolved regions in the reference genome.

Here, we introduce ContextSV, a novel SV detection method to identify large SVs that may be missed by current methods by integrating evidence beyond alignments, making it a valuable tool for improving sensitivity in SV detection pipelines. Our method increases performance for large copy number variants (CNVs) by integrating long-read alignments with coverage and SNV evidence information using a Hidden Markov Model (HMM) to predict SV copy number. We also incorporate a DBSCAN-based SV clustering method for efficient clustering of alignment evidence across a wide range of SV lengths. Finally, we develop ContextScore, a classification model that assigns SV confidence scores based on SV call and genomic context features, enabling the removal of low-confidence calls to improve precision of the final callset. We present a comprehensive evaluation of this method using real and simulated whole-genome SV benchmarking datasets, and present a case study of a few missed large CNVs in the KOLF2.1 cell line that we have experimentally validated.

## Materials and methods

### Implementation

ContextSV is implemented as a single C/C++ executable and optimized for speed and memory efficiency. HTSlib [7] is used to efficiently process alignments from long-read BAM files and variant records from the SNV VCF files. The copy number HMM is adapted from PennCNV [8] and PennCNV-Seq [9]. Multi-threading is used at chromosome-level workflow stages and in selected I/O operations.

From long-read whole-genome alignments, high-quality SNV VCFs and SNV population allele-frequency files, ContextSV generates an integrated structural variant callset using the following pipeline:

- HMM-based copy number predictions: We used the PennCNV-Seq [9] HMM trained to predict copy number states based on SNV B allele, population frequency and coverage from whole-genome sequencing data, and adapted it for the classification of CNVs from candidate SV breakpoints. The SNV B allele frequencies (obtainable from long-read SNV callers such as Clair3 [10] and NanoCaller [11]) and information about ethnicity-specific population frequencies (obtainable from the gnomAD database [12]) together provide information about observed and expected SNV frequencies, which are used as evidence for copy number changes. B-allele frequencies are derived from AD fields in the SNV VCF after quality filtering, and population frequencies are extracted from matched population VCFs. The coverage log2 ratio is an estimate of the normalized coverage at a given position in a chromosome and is calculated as the log2 ratio between the read depth at the SNV and the mean chromosome coverage across all non-zero depth positions. The HMM inference uses an adaptive sampling strategy rather than a fixed number of bins: the sample count scales with region size (approximately one observation per kb), with a minimum sampling floor of 20. SNV positions and B-allele frequencies (if found) are obtained, and coverage log2 ratios are calculated at each interval. The PennCNV [8] HMM C library is adapted to predict the expected sequence of copy number states given the SNV B allele, population frequency, and log2 ratio hidden state sequence comprising an SV candidate, which is then used for classifying large CNVs. Copy number state assignments are based on a dominant state fraction threshold, and predicted states are mapped to deletion, duplication, loss-of-heterozygosity (LOH), neutral, or unknown (no consensus). Data for all >10 kb SVs with copy number predictions, coverage log2 ratios, and SNV B-allele frequencies can be optionally saved in JSON format and as interactive plots for downstream validation and analysis.
- Merging direct alignment evidence: Structural variant candidate breakpoints are first obtained directly from alignment insertions and deletions by reading the BAM file CIGAR strings. To merge candidates for each SV type, we use DBSCAN with 1 − minimum reciprocal overlap as the epsilon distance parameter. This parameter measures the fraction of overlap relative to the smaller of the two intervals in proportion, and therefore decreasing epsilon from 1 increases strictness, clustering intervals only with high reciprocal overlap. By testing performance across multiple samples, we estimate an optimal epsilon = 0.1 with minPts = 5 for CIGAR alignment clustering, and 0.05 epsilon with minPts = 3 for split alignment clustering and the final combined clustering step. For each cluster, the merged SV is determined as the median length SV from the top 20% of the cluster. Finally, HMM-based copy number predictions are generated for each set of breakpoints that meet a minimum length threshold requirement (default 2 kb).
- Processing split alignment evidence: Primary alignments represent the best alignment for a read sequence, while supplementary alignments may provide evidence of larger, more complex events. Although there is a significant amount of alignment evidence, this evidence can be substantially merged by identifying consistent primary and supplementary alignment endpoints spanning the SV. We use the following approach to obtain a set of high-quality SV candidate breakpoints:
  - First, we efficiently cluster overlapping primary alignments using an interval tree.
  - For each primary-overlap group, we cluster primary start and end coordinates with 1D DBSCAN (epsilon = 100 bp, minPts = 5) and use the median of the largest cluster as merged endpoints. We similarly cluster supplementary endpoints and same-strand primary–supplementary read/reference distances, using largest-cluster medians as representative values.
  - Read and reference distances between 50 bp and 1 Mb are used as evidence for insertions (read > reference distance) and deletions (read < reference distance). The SV is called at the merged primary alignment positions using the distance as the SV length.
  - A primary cluster with majority alignments on opposite strands is classified as a potential inversion, with breakpoints defined by the merged supplementary endpoints at a maximum of 100 Mb in length.
  - Finally, HMM copy number predictions are generated for each SV breakpoint interval, and the SV is assigned a log likelihood for the predicted copy number sequence.
- Merging split alignment SV candidates: CIGAR-derived and split-derived candidates are clustered with DBSCAN and merged, keeping singletons. For clusters containing HMM-supported calls, the merge prefers the strongest HMM-supported representative according to implemented ranking logic. Final VCF writing applies additional quality filters, including resolving conflicts between alignment-based and copy-number-derived evidence, as well as optional filtering of SVs found in assembly gaps.

### Evaluations

For all evaluations, we compare performance versus Sniffles2 v2.4 [4], cuteSV v2.1.1 [5], and Delly (ONT mode) v1.3.1 [6]. To measure SV call performance relative to benchmark SV datasets, we use Truvari *bench* v5.3.0 [13]. SNV calls used in ContextSV copy number predictions were generated from long-read alignments using Clair3 v2.0.0 [10]. For all analyses, ContextSV calls were filtered with ContextScore.

### Datasets

**HG002**:

The HG002 SV benchmark dataset was downloaded from the Genome in a Bottle (GIAB) Consortium:

https://ftp-trace.ncbi.nlm.nih.gov/ReferenceSamples/giab/release/AshkenazimTrio/HG002_NA24385_son/NIST_SV_v0.6/

HG002 long reads sequenced with ONT R10.4.1 PromethION flow cells were obtained from an ONT public data release, basecalled with Guppy 6.1.2, and aligned to the GRCh37 reference genome with minimap2 v2.26-r1175: https://epi2me.nanoporetech.com/askenazi-kit14-2022-12/

**HG008**:

The recently released HG008 somatic SV benchmark dataset was downloaded from the Genome in a Bottle (GIAB) Consortium: https://www.nist.gov/programs-projects/cancer-genome-bottle

HG008 tumor and normal samples sequenced with PacBio HiFi Revio long reads aligned to the GRCh38 genome with 68× coverage for the normal sample and 106× coverage for the tumor sample were downloaded from GIAB:

https://ftp-trace.ncbi.nlm.nih.gov/ReferenceSamples/giab/data_somatic/HG008/Liss_lab/BCM_Revio_20240313

**Platinum Pedigree (NA12877, NA12878, NA12879)**:

The merged structural variant callset and ONT ultra-long sequencing reads for samples NA12877, NA12878, and NA12879 can be downloaded from the Platinum Pedigree Consortium: https://github.com/Platinum-Pedigree-Consortium/Platinum-Pedigree-Datasets

**KOLF2.1J iPSC**:

The previously published KOLF2.1J iPSC Illumina paired end short read whole genome sequencing (WGS) data were downloaded from the Alzheimer’s Disease Workbench: https://doi.org/10.34688/KOLF2.1J.2021.12.14

Our lab generated KOLF2.1J iPSC (at passage 3) ONT PromethION R10.4.1 30x long-read whole-genome sequencing data, followed by alignment to the GRCh38 reference genome using minimap2. Raw sequencing data have been deposited in the NCBI Sequence Read Archive (SRA) under BioProject accession number PRJNA1423767.

### Training the SV scoring/ranking model

ContextScore is a machine learning (ML) model trained on SV validation features and genomic context information to assign confidence scores to SVs in the final callset. These scores are used for filtering potential false positives to increase the precision of the final callset. ContextScore is trained on a set of 26 SV caller validation features and genomic context features (Supplementary Table S1). SV caller-based features include SV length, SV type, the type of alignment evidence used to call the SV, HMM copy number prediction likelihoods, read support, and breakpoint read depth. Genomic context features include segmental duplications (SDs), fragile sites, simple repeat regions, proximity to centromeres and telomeres, and highly conserved regions. Repetitive and low-complexity sequences such as SDs, fragile sites, and simple repeats confound read alignment and copy number inference, leading to spurious SV signatures and an elevated false positive burden that represents a persistent challenge in SV detection. In contrast, highly conserved regions are less prone to genuine structural variants, and calls arising from these regions are more likely to reflect alignment artifacts. Incorporating these annotations allows the model to learn the association between genomic context and call reliability, improving discrimination between true and false positive SVs across a range of challenging genomic contexts.

To train the ContextScore SV classification model, we used true-positive and false-positive SV calls from ContextSV across the Platinum Pedigree samples, HG002, and simulated long-read whole-genome benchmarking datasets. To avoid data leakage, each benchmarking dataset is evaluated using a model trained on all remaining datasets—yielding three held-out models: one trained on all data excluding HG002, one excluding the simulated dataset, and one excluding the Planstinum Pedigree samples. This leave-one-dataset-out strategy ensures that benchmark performance reflects generalization to unseen data rather than in-sample fit. A final model trained on all benchmarking datasets combined is used for deployment. We compute annotation-based genomic context features using a mixed pipeline: SD and cytoband annotations were obtained with ANNOVAR [14] (genomicSuperDups and cytoBand tracks), while fragile sites, conserved elements (phastCons100way), and simple repeats were annotated by BEDTools [15] intersections against BED files. We obtain a set of fragile site annotations from HumCFS, a manually curated database of human chromosomal fragile sites [16]. We obtain BED files for conserved and simple repeat regions from UCSC Table Browser resources [17].

We tested different ML models for binary classification including Logistic Regression, Random Forest, Support Vector Machine, and XGBoost. The full dataset for evaluating model performance includes 86 846 true positives and 62 311 false positives. We use a stratified 80/20 train-test split for benchmarking and handle imbalance during training with class-balanced modeling and SV length-aware sample weighting (50–500 bp, 500 bp–5 kb, 5–50 kb, and ≥50 kb bins). Hyperparameters are tuned by stratified cross-validation with precision as the optimization objective. In this setting, Random Forest achieved the highest test ROC–AUC (0.968) (Supplementary Fig. S1A) followed by XGBoost (0.960) (Supplementary Fig. S1B). Based on the Random Forest feature-importance analysis (Supplementary Fig. S2), the strongest contributors are cluster size per kb, same-type local SV count (especially 10 kb and 1 kb windows), and segmental-duplication overlap (left/right), followed by call evidence type and nearest same-type SV distance features. These results indicate that ContextScore relies on both local SV-density/context signals and read-evidence characteristics. Confidence-score distributions remain right-skewed with enrichment near zero (Supplementary Fig. S3). For filtering, we use adaptive SV type-specific probability thresholds estimated from each sample’s score distribution using a two-component Gaussian mixture model; if a given SV type had fewer than 20 variants or the fit was weak (BIC gain < 10, component gap < 0.08, separation < 1.0, or smaller component weight < 0.10), we used a fallback threshold of 0.2. The resulting threshold was then constrained to the 5–85th percentile range of the observed scores and capped at 0.3 to avoid overly aggressive filtering.

Additionally, we evaluate whether performance is consistent across the genome by performing per-chromosome cross-validation, leaving one chromosome for testing, and all other chromosomes for training. F1 for classification performance is fairly consistent (∼0.9) except for chromosome X (0.84), indicating the model can generalize across genomic contexts and is not overfitting to specific chromosomes (Supplementary Fig. S4). F1 is reduced on chrX consistent with known challenges, including pseudoautosomal regions (PAR1 and PAR2) and the X–transposed region (XTR) that are known sources of mapping and ploidy ambiguity [18, 19].

## Results

### Overview of the evaluations

**Figure 1.**
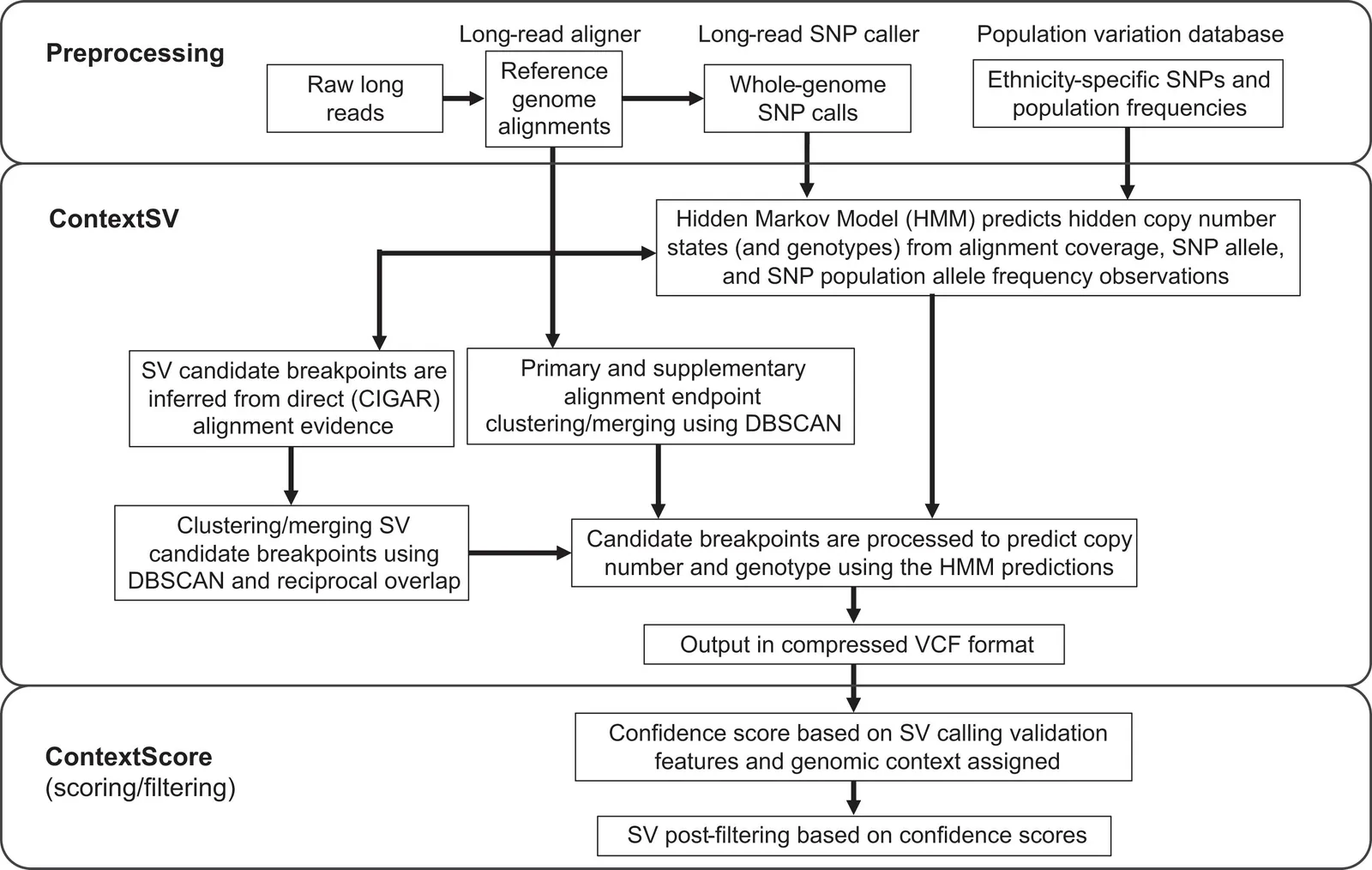
Overview of the ContextSV pipeline for SV detection from long-read sequencing data. Raw long reads are aligned to a reference genome, and whole-genome SNV calls are generated and annotated with ethnicity-specific allele frequencies from a population variant database. Within ContextSV, a HMM integrates alignment coverage, SNV B allele, and population frequency data to predict copy number states and genotypes. SV candidate breakpoints are identified from CIGAR and split alignment evidence, clustered, and merged using DBSCAN, then integrated with HMM predictions to generate consensus SV calls in compressed VCF format. Finally, ContextScore assigns a confidence score to each call based on validation features and genomic context, enabling post-filtering of the final SV callset.

An overview of the ContextSV method is shown in Fig. 1. We evaluate ContextSV performance for whole-genome SV calling using available benchmarking datasets including the GIAB HG002 SV v0.6 [20] and Platinum Pedigree datasets [21], which contain a set of high-confidence SV calls along with publicly available long-read sequencing data. SNV calls are generated from long-read alignments using Clair3 [10]. For all evaluations, we use Truvari [13] to compare performance versus widely used high-performance SV callers including Sniffles2 [4], cuteSV [5], and Delly [6].

Additionally, to ensure reliable evaluation of performance with a known ground truth dataset, we generate a simulated whole-genome SV and long reads dataset using VISOR [22]. This simulated dataset was generated based on gnomAD SVs from diverse populations [12]. We also evaluate our method’s performance for detection of somatic SVs from a paired tumor versus normal sample using the recently released draft HG008 benchmark dataset [23]. To assess SV genotyping accuracy, we use a trio from the Platinum Pedigree dataset and calculate the Mendelian error rate for SVs identified in the parents versus child.

Finally, to highlight the unique advantages of our method for detection of large SVs, we identify a few large >50 kb CNVs affecting genes in the KOLF2.1J cell line which were missed by other tools. We validate the presence of these CNVs using both long and short-read sequencing, followed by experimental validation of selected large SVs. We also include an evaluation on simulated ONT long-read sequencing data for a set of large clinically relevant inversions spanning several megabases (Mb) in length, to show performance of ContextSV relative to other tools in larger SV size intervals.

### Evaluation on HG002 from GIAB

We use the publicly available Genome in a Bottle (GIAB) SV benchmark dataset for the NA24385/HG002 sample from the Ashkenazi Jewish trio [20]. This benchmark provides a BED file of Tier 1 regions, defined as high-confidence regions where SV calls should be highly consistent across tools and that should contain near-comprehensive characterization of structural variants. This excludes regions with discordant calls from their analysis of multiple variant callers and therefore excludes many complex repetitive regions. The dataset contains 12 745 SVs, which are reduced to 9646 after filtering by Tier 1 regions in Truvari (Supplementary Fig. S5).

In the HG002 SV benchmarking dataset from GIAB, there are only five deletions and one insertion with lengths >50 kb (Supplementary Fig. S5), and therefore evaluation of performance for large SVs is limited with this dataset. In summary, from the article, the following are the stated classes of structural variants that cannot be evaluated with this benchmark dataset [20]:

- Insertions, deletions, and large CNVs > 100 kb (only one insertion and deletion >100 kb)
- Inversions, translocations, and non-tandem duplications
- SVs in SDs and in >10 kb tandem repeats

Across all SV types, cuteSV achieved the highest F1 score (0.962) and recall (0.971), followed closely by Sniffles2 (F1: 0.955; recall: 0.963) and Delly (F1: 0.952; recall: 0.975), with Delly achieving the highest raw recall overall (Table 1). All four callers produced comparable total SV counts (∼9600–9900). While ContextSV exhibits reduced precision (0.873) and recall (0.907) in standard benchmark conditions, it should be noted that GIAB benchmarks are predominantly composed of well-characterized, high-confidence SVs in accessible genomic regions, and performance on this benchmark may not fully capture caller behavior in more challenging contexts such as somatic or repeat-rich variation.

**Table 1.** Comparison of ContextSV performance versus widely used long-read SV callers on ONT HG002 data using the GIAB v0.6 benchmark dataset

NA24385 (HG002) GIAB SV v0.6 benchmark							
SV caller	SV count	F1	Precision	Recall	TP	FP	FN
ContextSV	9819	0.89	0.873	0.907	8576	1243	880
Sniffles2	9619	0.955	0.947	0.963	9108	511	348
cuteSV	9635	0.962	0.953	0.971	9178	457	278
Delly	9923	0.952	0.930	0.975	9224	699	232

SV caller versions are Sniffles2 v2.4, cuteSV v2.1.1, and Delly v1.3.1. Results were generated using Truvari.

We further perform a benchmarking study stratifying by SV type and size (Supplementary Fig. S6). Performance diverges at larger intervals: ContextSV achieves high recall for >10 kb SVs, with a trade-off in precision for 10–50 kb SVs, most notably for duplications/insertions. Both ContextSV and cuteSV achieve F1 of 1.0 for 50–100 kb SVs (0.8 for Sniffles2 and Delly), although there are only three SVs in this interval, therefore an advantage for SVs in this interval cannot be well determined. Although there is only one insertion and deletion >100 kb (Supplementary Fig. S5), a limitation in this benchmark, all callers except cuteSV identify the deletion. These observations should be interpreted cautiously given the low variant counts at higher size intervals, which can inflate metric variability.

Improved recall at >10 kb size intervals relative to alignment-based methods is expected, particularly for large CNVs, due to the integration of copy number predictions with long-read alignment evidence. Precision remains a challenge for ContextSV specifically for 10–50 kb SVs. Nevertheless, this dataset is limited to high-confidence regions with a limited range of SV size and complexity (Supplementary Fig. S5). Therefore, the evaluation is a limited reflection of actual performance for this method, particularly for detection of large SVs and SVs in more challenging regions of the genome. This necessitates further evaluation with datasets containing a more comprehensive set of whole-genome structural variants.

### Evaluation on a Platinum Pedigree Trio

The Platinum Pedigree study used PacBio and ONT long read sequencing and multiple long-read-based and assembly-based SV callers to obtain a merged SV truth set for CEPH-1463 pedigree samples [21]. This callset includes deletions, insertions, and inversions. Here, we analyze benchmarking results for a trio from this dataset: parents NA12877 (24 160 SVs) and NA12878 (24 171 SVs), and child NA12879 (24 338 SVs). Although this dataset contains larger SVs than the GIAB benchmark, it contains only a few SVs > 50 kb (22 for NA12877, 21 for NA12878, and 22 for NA12879), and no SVs > 100 kb (Supplementary Fig. S5). In Table 2, we summarize whole-genome benchmarking results for samples across SV types. Consistent with the HG002 benchmark, ContextSV showed lower overall F1 scores (0.628–0.638) relative to Sniffles2 (0.762–0.769), cuteSV (0.776–0.787), and Delly (0.728–0.737) across all three samples. This gap is primarily driven by substantially higher false positive counts in ContextSV (11 878–12 850) compared to the other callers (4964–8825), reflecting reduced precision (0.565–0.579). Notably, however, ContextSV’s recall was broadly comparable to the other callers across all three samples (0.686–0.709), and in some cases approached that of Delly, which achieved the highest recall overall. ContextSV also produced the largest SV callsets (28 165–29 526 SVs), which likely contributes to both its higher false positive rate and its maintained recall. While ContextSV’s overall benchmark performance lags behind other callers in these samples, its recall consistency across all three samples suggests it is not systematically missing true SVs, and the elevated false positive rate may in part reflect sensitivity to larger SVs not well-represented in the benchmark truth set.

**Table 2.** Comparison of ContextSV performance versus widely used long-read SV callers on ONT ultra-long sequencing read alignments using the NA12877, NA12878, and NA12879 SV benchmarks from the Platinum Pedigree dataset

Platinum Pedigree benchmark evaluations								
Sample	SV caller	SV count	F1	Precision	Recall	TP	FP	FN
NA12877	ContextSV	28 793	0.638	0.579	0.709	16 682	12 111	6851
NA12877	Sniffles2	23 912	0.762	0.756	0.768	18 068	5844	5465
NA12877	cuteSV	23 932	0.776	0.769	0.782	18 414	5518	5119
NA12877	Delly	27 352	0.728	0.677	0.787	18 527	8825	5006
NA12878	ContextSV	29 526	0.628	0.565	0.707	16 676	12 850	6896
NA12878	Sniffles2	23 749	0.763	0.760	0.765	18 042	5707	5530
NA12878	cuteSV	23 635	0.779	0.778	0.780	18 393	5242	5179
NA12878	Delly	27 434	0.733	0.681	0.793	18 683	8751	4889
NA12879	ContextSV	28 165	0.628	0.578	0.686	16 287	11 878	7453
NA12879	Sniffles2	24 143	0.769	0.762	0.775	18 408	5735	5332
NA12879	cuteSV	23 591	0.787	0.790	0.785	18 627	4964	5113
NA12879	Delly	27 144	0.737	0.691	0.790	18 758	8386	4982

Results were generated using Truvari.

Finally, we perform a benchmarking study stratifying by SV type and size for NA12877 (Supplementary Fig. S7), NA12878 (Supplementary Fig. S8), and NA12879 (Supplementary Fig. S9). There is a sharp drop-off in performance across callers for >10 kb SVs. ContextSV exhibits high recall for >5 kb SVs consistently across samples, with variable-to-low performance for <5 kb SVs. Due to the small number of inversions in this benchmark, performance is variable across callers and samples and cannot be well assessed (Supplementary Fig. S5).

**Figure 2.**
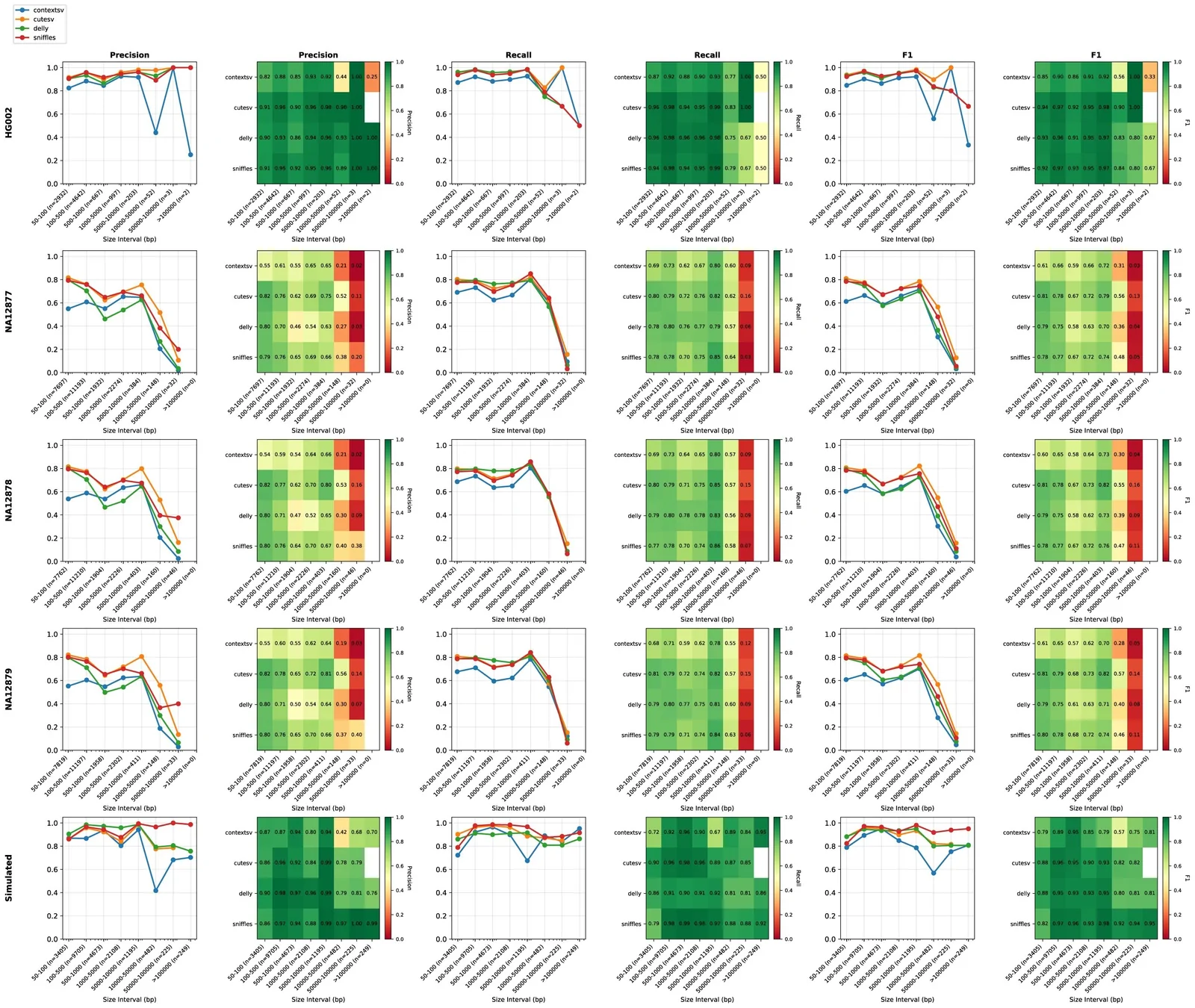
Performance metrics across SV benchmarking datasets (HG002, Platinum Pedigree, and simulated dataset). Here, we compare F1, precision, and recall across all SV types, stratified by size intervals.

This benchmark does not include a curated list of high-confidence regions for SV detection; therefore, it is expected that overall whole-genome benchmarking performance will be low in comparison to the HG002 GIAB benchmark results filtered for Tier 1 high-confidence regions (Fig. 2). We found that performance is increased across callers when filtering for Tier 1 regions (lifted over to GRCh38) similar to the previous HG002 evaluation (Table 3). The limited range of SV size and type in this benchmark dataset necessitated further evaluation with simulated data.

**Table 3.** Comparison of ContextSV performance versus widely used long-read SV callers on ONT ultra-long sequencing read alignments in GIAB SV v0.6 Tier1 high-confidence regions for the NA12877, NA12878, and NA12879 SV benchmarks from the Platinum Pedigree dataset

Platinum Pedigree benchmark evaluations (GIAB SV v0.6 Tier 1 regions)								
Sample	SV caller	SV count	F1	Precision	Recall	TP	FP	FN
NA12877	ContextSV	10 875	0.845	0.847	0.844	9206	1669	1706
NA12877	Sniffles2	10 566	0.917	0.932	0.903	9850	716	1062
NA12877	cuteSV	10 593	0.925	0.939	0.911	9946	647	966
NA12877	Delly	10 942	0.909	0.908	0.911	9938	1004	974
NA12878	ContextSV	10 929	0.842	0.842	0.842	9200	1729	1721
NA12878	Sniffles2	10 569	0.916	0.931	0.901	9842	727	1079
NA12878	cuteSV	10 579	0.924	0.939	0.910	9937	642	984
NA12878	Delly	10 968	0.912	0.910	0.914	9980	988	941
NA12879	ContextSV	10 701	0.836	0.848	0.825	9070	1631	1928
NA12879	Sniffles2	10 734	0.915	0.926	0.904	9945	789	1053
NA12879	cuteSV	10 673	0.922	0.936	0.908	9991	682	1007
NA12879	Delly	11 028	0.910	0.909	0.911	10 020	1008	978

Results were generated using Truvari.

### Evaluation on a simulated long read SV benchmark dataset

To generate a ground truth dataset of simulated SVs for benchmarking, we use VISOR, which simulates ONT sequencing reads and alignments from a reference genome and a predefined set of SVs [22]. To construct a simulated whole-genome dataset, we sample a set of known SVs from the gnomAD v4 dataset, which contains ∼1.2 million high-quality SVs identified from 63 046 whole-genome sequencing samples from diverse populations, and which were validated using both short and long-read sequencing methods [12]. To obtain a representative whole-genome SV dataset across a wide range of lengths based on the gnomAD SVs, for each chromosome we sample the first 500 insertions and deletions within the size range 50 bp–10 kb, followed by the first 4 insertions, deletions, inversions, and duplications in the size intervals 10–20, 20–50, 50–100, and 100–600 kb (if any). After filtering overlapping SVs, this resulted in 21 957 whole-genome SVs, comprising 10 686 deletions, 10 919 combined insertions and duplications, and 352 inversions. Of these there are 940 SVs > 10 kb, 473 SVs > 50 kb, and 249 SVs > 100 kb, with a maximum SV size of 572 kb (Supplementary Fig. S5).

ContextSV exhibits low overall F1 (0.877) compared to the other callers, driven by a high false positive count (3045) relative to Sniffles2 (751), cuteSV (948), and Delly (734) (Table 4). ContextSV also produced a higher total call count (22 572) than the other callers (∼20 000–21 000). These results motivate future work on improving call-level filtering to reduce false discoveries.

**Table 4.** Comparison of ContextSV performance versus widely used long-read SV callers on a simulated SV dataset

Simulated SV benchmark							
SV caller	SV count	F1	Precision	Recall	TP	FP	FN
ContextSV	22 572	0.877	0.865	0.889	19 527	3045	2430
Sniffles2	21 356	0.951	0.965	0.938	20 605	751	1352
cuteSV	21 460	0.945	0.956	0.934	20 512	948	1445
Delly	20 291	0.926	0.964	0.891	19 557	734	2400

Results were generated using Truvari.

**Figure 3.**
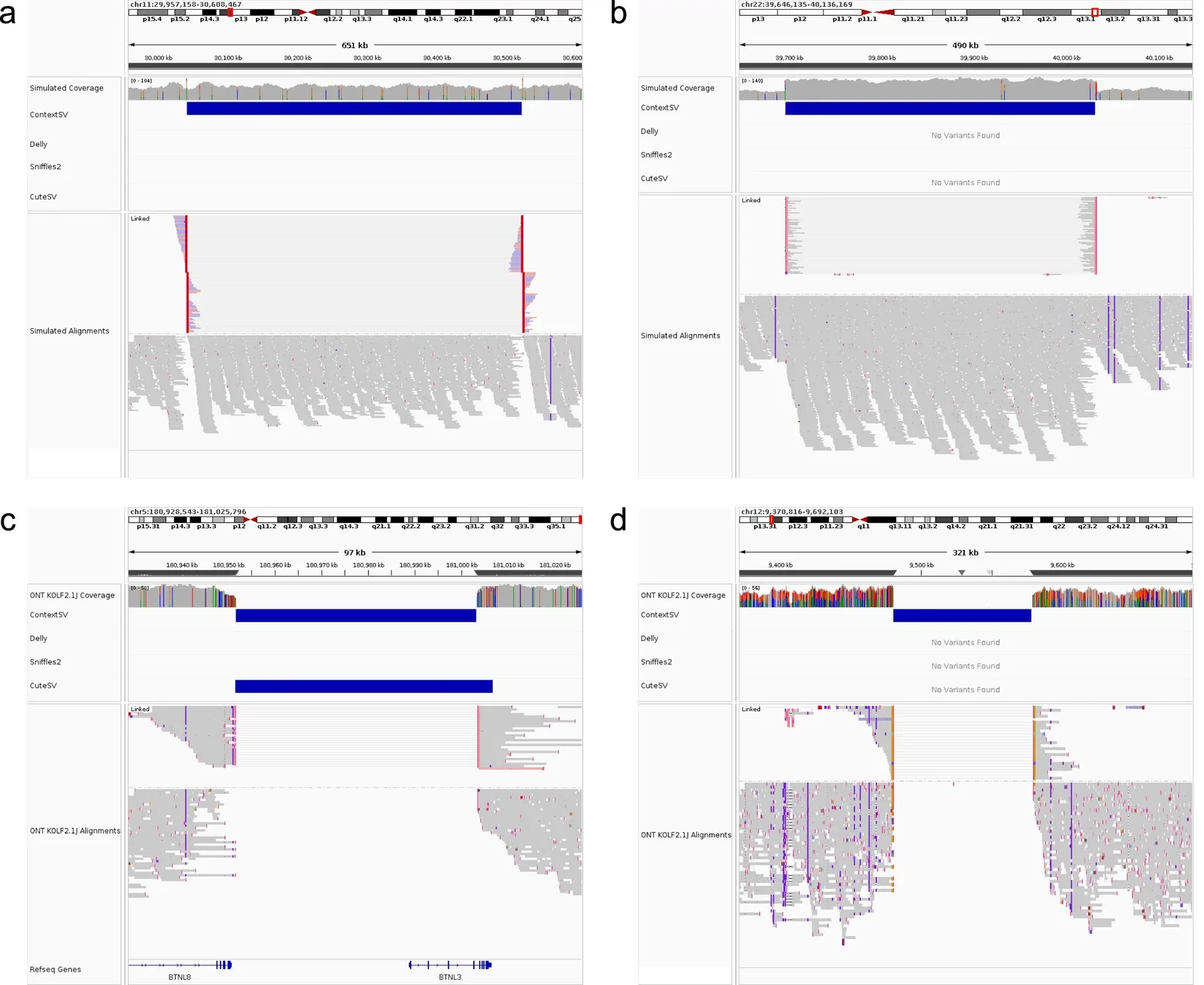
A few examples of large SVs identified by ContextSV but not identified by most callers. ONT long read alignments are shown in IGV for the simulated dataset (**a** and **b**) and for KOLF2.1J (**c** and **d**). (A) A 482 kb inversion identified only by ContextSV in the simulated dataset. (B) A 336 kb duplication identified only by ContextSV in the simulated dataset. (C) A 51 kb homozygous deletion on BTNL3 identified only by ContextSV and cuteSV in KOLF2.1J from ONT long-read sequencing. (D) A 99kb homozygous deletion identified only by ContextSV in KOLF2.1J from ONT long-read sequencing.

We perform a further benchmarking study stratifying by SV type and size (Supplementary Fig. S10). In this analysis, our simulated ground truth benchmark dataset allows us to separately evaluate insertion and duplication performance. Consistent with previous results, ContextSV shows high recall for >10 kb deletions and duplications, with a trade-off in precision. ContextSV and Sniffles2 both exhibit high performance for >10 kb inversions, although Sniffles2 exhibits higher precision. For <10 kb SVs, inversion calling was highly variable across all callers and size intervals, although Sniffles2 exhibited consistent high performance for >1 kb size intervals. ContextSV exhibited low overall performance for insertions, representing a key area for future improvement. In Fig. 3, we show an example of a 482 kb inversion (Fig. 3A) and a 336 kb duplication (Fig. 3B) from this dataset identified only by ContextSV. We find that this is mostly due to long-read SV callers using a split alignment distance threshold to reduce false positives, while ContextSV does not require this. ContextSV integrates clustered split alignment breakpoint evidence with HMM log likelihood evidence to identify a true inversion call in this case. Taken together, these results indicate that while ContextSV exhibits high recall for multiple SV types at large size intervals, filtering false positives at larger size intervals represents a key focus of future improvements.

### Evaluation of somatic SV detection on HG008 from GIAB

**Figure 4.**
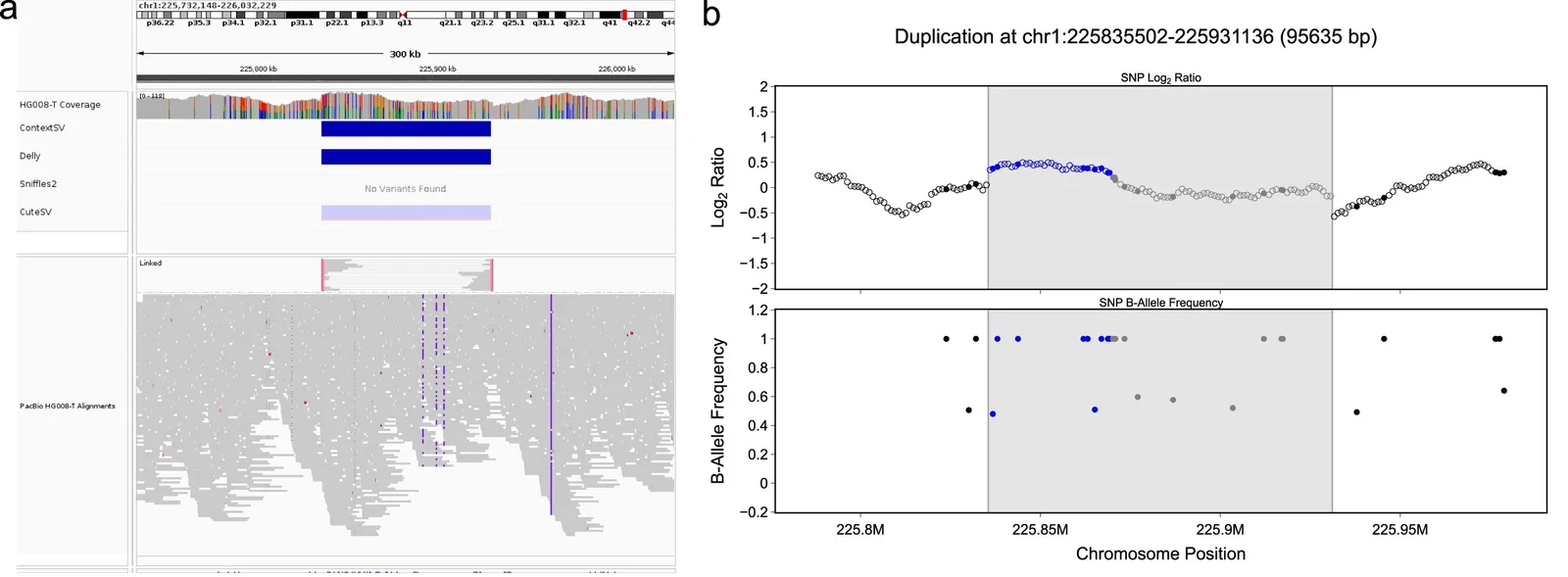
PacBio HiFi long-read alignments shown in (**a**), IGV for a large ∼95 kb deletion from the HG008 draft somatic SV benchmark dataset which was identified by ContextSV and Delly but missed by Sniffles2 and filtered by cuteSV. Shown in (**b**) is the ContextSV HMM copy number prediction evidence, with the top panel showing coverage log_2_ ratio and the bottom panel showing SNV BAF (blue = duplication prediction, gray = neutral copy number prediction, vertical line = Breakpoints determined from long-read alignments, filled circle = SNV + coverage evidence used, open circle = only coverage evidence used.

The identification and characterization of somatic structural variants is of high importance, particularly in human cancer genomes. The Genome in a Bottle (GIAB) Consortium recently released a draft benchmarking dataset for somatic SV detection with long-read whole genome sequencing data of paired tumor-normal cell lines for the HG008 sample [23]. This dataset can be used to evaluate the performance of somatic SV callers at distinguishing between somatic SVs (present in the tumor but not in the germline) and germline mutations (inherited genetic variants). We use publicly available PacBio HiFi Revio long read sequencing data aligned to the GRCh38 genome with 68× coverage for the normal sample and 106× coverage for the tumor sample, and identify SVs using ContextSV, Sniffles2, cuteSV, and DELLY. We use Truvari to identify somatic SVs by comparing tumor vs. normal to obtain somatic SV calls. We then compare these somatic SVs to the draft benchmark dataset. We find that ContextSV identifies a comparable number of somatic SVs relative to other tools (Table 5). Of these, a large 95 kb duplication was only found by ContextSV and Delly (ONT mode), with a filtered call from cuteSV (Fig. 4). Duplications are more challenging to identify from alignment breakpoint evidence alone, but ContextSV can utilize coverage and SNV evidence to accurately classify the duplication.

**Table 5.** Comparison of ContextSV performance versus widely used long-read SV callers on PacBio HiFi data using the HG008 somatic SV benchmark dataset from GIAB

SV caller	SV type	F1	Precision	Recall	TP	FP	FN	Somatic SV count
ContextSV	Deletions	0.053	0.028	0.606	20	706	13	1194
Sniffles2	Deletions	0.179	0.099	0.970	32	292	1	716
cuteSV	Deletions	0.171	0.094	0.939	31	299	2	721
Delly	Deletions	0.102	0.054	1.000	33	581	0	1124
ContextSV	Dup./Ins.	0.046	0.024	0.755	40	1630	13	2374
Sniffles2	Dup./Ins.	0.138	0.075	0.906	48	594	5	1080
cuteSV	Dup./Ins.	0.016	0.009	0.208	11	1282	42	1817
Delly	Dup./Ins.	0.112	0.059	1.000	53	840	0	1317

SV comparisons were performed using Truvari bench.

Somatic SV detection performance varies across callers and SV types. Precision was uniformly low across all callers (0.02–0.1), which is expected for this stage of analysis: somatic candidates were derived via tumor-normal subtraction without additional post-filtering, and published benchmarks of somatic SV calling report similar false-positive burdens prior to allele frequency or multi-caller filtering [24]. Recall is, therefore, the more informative metric at this stage, as it reflects the upper bound of true somatic SVs recoverable before downstream refinement.

Among callers, Sniffles2 consistently achieved the best balance of recall and callset size for both deletions (recall = 0.97; 716 somatic calls) and duplications/insertions (recall = 0.91; 1 080 somatic calls). Delly achieved perfect recall for both SV types but at the cost of the highest false-positive burden (581 and 840 FP, respectively), suggesting permissive rather than specific calling. cuteSV performed competitively for deletions (recall = 0.94) but nearly failed for duplications and insertions (recall = 0.21). ContextSV exhibited lowest recall for deletions but demonstrated stronger performance for duplications and insertions (recall = 0.76; 40/53 TP). In summary, ContextSV recovers a non-trivial number of true positive insertions and duplications (40/53), some which may not recovered by other callers. The complementary recall profiles across callers suggest that ensemble approaches combining multiple callers may improve both sensitivity and specificity for comprehensive somatic SV detection.

### Evaluation of genotyping accuracy on Platinum Pedigree

Trio-based analysis of Mendelian inconsistencies is a good method for evaluating false positive SV calls from whole-genome sequencing data, since the *de novo* mutation rate for offspring is very low [25]. To evaluate Mendelian inconsistencies, we compare SV callsets for NA12879 with the parents NA12877 and NA12878. For SV comparisons, we used Truvari with parameters *pctovl *= 0.5, *pctsize *= 0.5, and *refdist *= 1000. To calculate Mendelian error rate in NA12879, we find the union of the parent SVs, identify SVs unique to NA12879, and then divide by total NA12879 SVs.

Mendelian error rates varied considerably across SV types and callers (Table 6). ContextSV showed a high duplication error rate (32.48%) relative to cuteSV (17.46%) and Delly (17.49%), while Sniffles2 showed a markedly elevated duplication error rate (75.00%), suggesting substantial challenges in accurate duplication genotyping across long-read callers generally. ContextSV achieved an error rate of 21.54% for inversions, outperforming cuteSV (40.30%) but trailing Delly (18.68%) and Sniffles2 (13.33%). Overall, while ContextSV’s aggregate Mendelian error rate (14.42%) was somewhat higher than the other tools (ranging from 8.85% to 9.95%), this may reflect broader and more sensitive SV detection, particularly for duplications and inversions, where other callers may underreport or miscall variants.

**Table 6.** Comparison of percent Mendelian error rate in ContextSV versus widely used long-read SV callers stratified by structural variant (SV) types using a trio from the Platinum Pedigree dataset

SV type	ContextSV	Sniffles2	cuteSV	Delly
All	14.42	9.95	8.85	9.9
Deletions	14.29	9.02	5.68	10.93
Insertions	13.94	10.59	11.21	8.69
Duplications	32.48	75	17.46	17.49
Inversions	21.54	13.33	40.3	18.68

Interpretation of Mendelian error rates should be considered alongside the underlying SV call sets (Table 7). ContextSV produced a moderately sized callset (42 690 merged parent SVs; 29 404 child SVs) with 4 240 child-unique SVs—those present in the child but absent in either parent, which form the basis of the Mendelian error calculation. Notably, lower Mendelian error rates do not straightforwardly indicate superior performance; large or permissive callsets such as Delly’s (69 808 merged parent SVs) can yield low aggregate error rates while underrepresenting large or complex SVs. The number of SVs unique to the child (not present in either parent) can also be inflated either due to false negatives (missed SVs) in the parents, or false positives in the child. Finally, false negatives affecting inherited/shared SVs in the child is also a source of inflated Mendelian error rate.

**Table 7.** Structural variant callset counts for merged parent (NA12877 and NA12878), child (NA12879), and child unique SVs from the Platinum Pedigree dataset

SV caller	Merged parent SV count	Child SV count	Child unique SV count
ContextSV	42 690	29 404	4240
Sniffles2	36 911	24 993	2494
cuteSV	56 303	33 631	2978
Delly	69 808	41 561	4119

SV comparisons were performed using Truvari bench.

### Evaluation on the KOLF2.1J cell line

KOLF2.1J is a cell line that is being prioritized as a reference standard for studying neurodegenerative diseases, which necessitates a more complete structural characterization of its genome to understand variant effects. A complete telomere-to-telomere assembly and initial characterization of structural variants in KOLF2.1J has been recently completed [26]. In a previous study, we used the Delly SV caller together with coverage and SNV allele frequency evidence to validate the presence of a few large exonic CNVs affecting genes of functional importance in the KOLF2.1J iPSC cell line from Illumina short read whole genome sequencing data [27]. Long-read sequencing-based analysis of structural variants in the KOLF2.1J cell line allows us to investigate the sensitivity of all the SV callers on detecting a verified large SV from a real sample as opposed to synthetic data. To accomplish this, we sequenced the KOLF2.1J cell line using ONT PromethION R10.4.1 with 30× coverage, followed by alignment to the GRCh38 reference genome using minimap2 [28] and SV calling using ContextSV, Sniffles2 [4], cuteSV [5], and Delly [6] on the KOLF2.1J long-read data.

**Figure 5.**
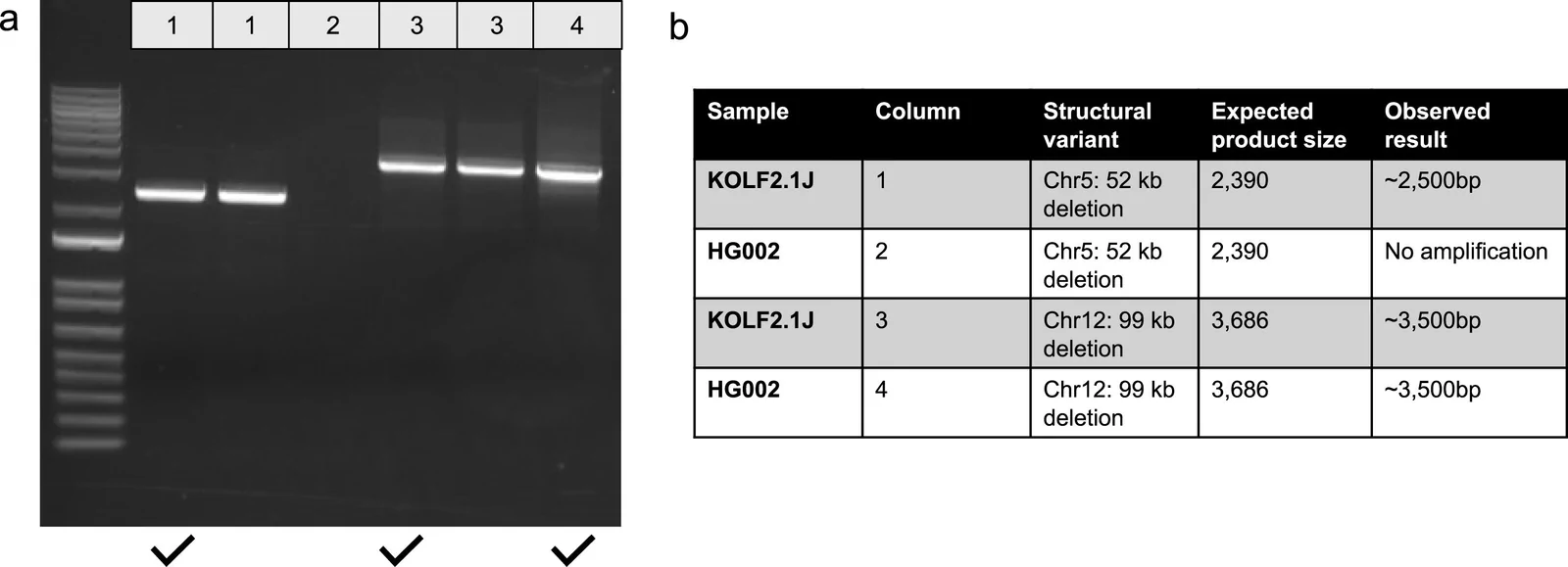
PCR and Sanger validation of large structural variants. (**a**) Agarose gel electrophoresis showing PCR products for deletions in KOLF2.1J and HG002. Lane 1: chr5 deletion in KOLF2.1J (∼2.5 kb); Lane 2: chr5 deletion in HG002 (no product); Lanes 3–4: chr12 deletion in KOLF2.1J and HG002 (∼3.5 kb). (**b**) Summary of expected vs observed amplicon sizes across the two samples.

We identify a few large SVs that were not identified by most tools, including a 51 kb homozygous exonic deletion on chr5 BTNL3 identified only by ContextSV and cuteSV (Fig. 3C), and a 99 kb homozygous deletion on chr12 identified only by ContextSV (Fig. 3D). Validation of these SVs in KOLF2.1J was performed using PCR with primers designed to amplify across breakpoint junctions, and products were sequenced by Sanger to confirm the predicted breakpoint sequences. We use HG002 as a reference sample for this validation. Using this approach, we validated the presence of the two deletions in KOLF2.1J (Fig. 5). From this validation, the chr5 deletion was absent in HG002, whereas the chr12 deletion was present, consistent with ContextSV calls on HG002. These SVs were missed by other callers due to filtering steps during calling, including limits for maximum split alignment distance, which can be high for SVs with breakpoint ambiguity. ContextSV has a unique advantage in this case since it utilizes copy number predictions as supplementary evidence, enabling a confident call for SVs using HMM log likelihood. These findings highlight the utility of this method at identifying large CNVs which may be missed by current methods, making it an important complementary tool in SV detection pipelines.

### Evaluation on large simulated clinically relevant inversions

Previous analysis shows robust performance of ContextSV in larger SV size intervals, and we evaluated whether this advantage holds for SVs that span several megabases (Mb) in length. There are several rare disease patients reported with non-coding diseases that carry clinically relevant inversions that span Mbs, including a 12.54 Mb inversion in patient with Axenfeld–Rieger syndrome (ARS) [29], F-syndrome patient with 1.1 Mb inversion [30], Branchiooculofacial syndrome (BOFS) patient with 88.75 Mb inversion [31], Microphtalmia patient with a 3.16 Mb inversion [32], and a Craniofrontonasal syndrome patient with a 2.01 Mb inversion [33]. We use VISOR [22] to simulate ONT sequencing reads and alignment data for this set of inversions and calculate reciprocal overlap for the identified inversion (Table 8). ContextSV and Sniffles2 successfully identify all five large inversions with near-1.0 reciprocal overlap, while cuteSV and Delly miss all five inversions.

**Table 8.** Evaluation of ContextSV and other widely-used long-read SV callers on identification of a set of large Mb-level clinical inversions with ONT data simulated using VISOR

Inversion location	Inversion length (Mb)	ContextSV reciprocal overlap	Sniffles2 reciprocal overlap	cuteSV reciprocal overlap	Delly reciprocal overlap
chr6:10355280–99103873 (hg19)	88.75	1	1	N/A	N/A
chrX:68167790–70181179 (hg19)	2.01	0.999999	1	N/A	N/A
chr2:219741632–220839811 (hg19)	1.10	0.999996	0.999998	N/A	N/A
chr4:98141399–110685792 (hg38)	12.54	1	1	N/A	N/A
chr6:1713354–4870965 (hg38)	3.16	1	0.999999	N/A	N/A

Reciprocal overlap is calculated for the best candidate structural variant, with N/A indicating the inversion was not identified by the caller.

### Unique advantages for SV detection

In the previous sections, we describe a few instances where real large SVs were missed by other methods in KOLF2.1J, HG008, and in simulated datasets. To highlight the unique advantages of our method, we identified the source of the missed SVs for each evaluated algorithm (Sniffles2, cuteSV, and Delly) for the HG008 95kb duplication. For Sniffles2, the duplication was removed during calling due to exceeding a threshold based on relative coverage around the SV. For cuteSV, the SV was filtered due to low (<5) read support, due to insufficient clustering of breakpoint evidence from split alignments. By troubleshooting SVs missed by other callers, it is clear that differences in SV calls occur due to differences in read support for SVs obtained from clustering alignment evidence, and due to differences in filtering approaches used for improving precision. ContextSV uses HMM copy number predictions to calculate a log likelihood for SVs, which is integrated with alignment evidence to classify this duplication (Fig. 4A and B). ContextSV also uses DBSCAN for clustering breakpoints with reciprocal overlap as the distance parameter, resulting in improved clustering of read alignment evidence for large SVs. Long-read SV callers typically utilize filtering approaches to identify and filter potential false positive SV candidates based on read support and other alignment features, such as local SV coverage and split alignment distances on the read versus reference genome. Alignment evidence is sparser for larger SVs since the read lengths are insufficient to capture the full event, and therefore breakpoints are inferred from split alignment evidence. Clustering split alignment evidence to identify candidate breakpoints is more challenging for genomic regions more prone to alignment errors, such as SDs, centromeres, and satellite repeats. Our method can identify large SVs while minimizing duplicate false positive calls, which is accomplished by integrating copy number predictions and utilizing a novel SV clustering approach that is effective for identifying breakpoints from split alignments in larger SVs and in challenging genomic regions.

## Discussion

The increased read lengths and accuracy of the latest long-read sequencing technologies allow for the development of novel computational methods that can leverage these advantages and obtain a more comprehensive picture of human genomic structural variants. ContextSV is a long-read SV caller that analyzes long-read alignments together with SNV allele and coverage information to identify and genotype large SVs which may be missed by current major long-read SV callers.

In summary, long-read alignment-based SV callers follow similar approaches for identifying SV candidates but tend to rely on the quality of alignment evidence, which tends to limit the size and complexity of SVs, and also introduces challenges in identifying and filtering false positives for both PacBio and ONT data [34]. Due to the unique approaches of each long-read SV caller, it is often advised to use an ensemble approach combining results from multiple algorithms to obtain a more comprehensive callset [35].

In general, current widely used long-read SV calling methods identify breakpoints from either reads or read assemblies aligned to a reference genome [3]. The reference genome provides a standard coordinate system and sequence for aligning reads. Major long-read aligners include Minimap2 [28], NGMLR [36], and BLASR [37] seed and chain together matching sequences in the reads versus reference to yield a summary of operations including matches, mismatches, insertions, deletions, and strand information [38]. This information is integrated by long-read SV callers to classify different types of structural variants. The major long-read sequencing platforms such as ONT and PacBio have unique characteristics in their sequencing data including in read length distributions, accuracy, and coverage. These differences across sequencing platforms influence the quality of alignments and are all features long-read SV callers must consider. Major alignment-based long-read SV callers including Sniffles2 [4], cuteSV [5], and SVIM [39] provide support for the unique long read features of both PacBio and ONT. There are also callers specific to long-read sequencing platforms, such as PBSV designed for PacBio long reads (https://github.com/PacificBiosciences/pbsv), as well as NanoSV [40] and NanoVar [41] for ONT data. Additionally, well-established short-read SV callers such as Delly [6] now provide support for long-read SV calling with PacBio and ONT data as well.

Current widely used long-read SV callers such as Sniffles2 [4] and cuteSV [5] are alignment-based, meaning that SVs are identified directly from read alignments to a linear reference genome. Insertions, deletions, and split-read alignments are used to identify and classify candidate SVs. Split reads occur when there is more than one potential read alignment identified by the aligner. Evidence from split reads can be used to identify large SVs that surpass the length of individual reads. Additionally, supplementary alignments can provide important evidence for inversions if the alignments map to opposite strands, or inter-chromosomal translocation events if the alignments map to different chromosomes. Sniffles2, cuteSV, and Delly all utilize heuristic approaches to compare differences in distance and orientation between primary and supplementary alignments on the read versus on the aligned reference genome coordinates. This information is combined with breakpoint evidence from direct alignments to classify SV types including insertions, deletions, duplications, inversions, and translocations. Insertions, deletions, and soft clips in the direct alignment provide evidence for SV breakpoints, and distance between split alignments on the read versus reference genome on the same strand indicate potential deletions (small read versus reference distance) or duplications (large read versus reference distance). Similarly, orientation differences and inter-chromosomal alignments are used to classify potential inversions and translocations.

This compilation of read alignment evidence is typically followed by a computationally efficient clustering method. Clustering read alignment evidence is important for streamlining downstream SV classification tasks that rely on more sophisticated and computationally intensive algorithms. Sniffles2 performs clustering by binning to identify SV candidates from alignment evidence and merges candidates based on SV genomic position, type, and length. cuteSV utilizes a heuristic method to merge smaller alignments into larger consensus SV candidates based on genomic distances between candidates, followed by a similar clustering by binning, and merging based on SV type and genomic distance. Delly similarly merges final SV candidates of the same type by utilizing an absolute distance threshold between breakpoints. One potential limitation of clustering using binning is that there is a potential trade-off of bin size with breakpoint resolution. If using a small bin size, high accuracy breakpoints can be obtained for smaller SVs but with a potential loss of long-range breakpoint evidence for larger SVs.

SV discovery is followed by genotyping, which is the process of determining the presence or absence of SV alleles in an individual, including homozygous versus heterozygous reference versus alternate alleles [42]. This is typically estimated from calculating the likelihood of possible genotypes based on read support for reference versus alternate alleles. Sniffles2 predicts the most probable SV genotype by calculating the maximum likelihood of different possible genotypes (e.g. homozygous reference [0/0], heterozygous [0/1], and homozygous variant [1/1]) from a binomial distribution of read counts supporting variant and reference alleles. cuteSV computes a likelihood score for each genotype based on read support for reference versus alternate allele breakpoints within each SV candidate cluster. The genotype is then determined from the maximum likelihood genotype. Delly’s genotyping process similarly works by extracting read support for reference and alternative alleles at SV breakpoints, computing read alignment scores for the alleles based on edit distances, and finally using these scores to obtain a genotype based on read support for reference vs. alternate alleles. This approach relies on the distributions of read alignments for each allele accurately reflecting the genotype and therefore remains limited by the alignment quality.

Finally, SV candidates undergo a post-filtering step based on important validation features, typically using fixed thresholds for minimum read depth, read alignment support, and cluster size. Without integration of additional evidence, it is challenging to identify large SVs from split alignment evidence with high precision. Therefore, additional filtering criteria are used, such as a maximum distance on the read for split alignments, minimum mapping quality scores, a minimum read depth in the center of the SV (Sniffles2), a local coverage-based threshold (Sniffles2), or simply a maximum SV length (cuteSV). Long-read SV callers such as NanoSV [40] and NanoVar [41] employ a ML-based method trained on alignment features to score SVs and filter potential false positives. ML-based methods can consider many features simultaneously, learning their joint contribution to true versus false positive SVs to yield more optimal decision boundaries for filtering. For example, a true SV in a SD region may show low mapping quality, which can be offset by high read depth and split alignment evidence for breakpoints. Genomic context is also important for SV validation, as regions such as SDs, centromeres, or satellite repeats are more prone to alignment errors [43]. GATK-SV, an ensemble pipeline for short-read whole-genome SV calling, uses an ML model that incorporates genomic context, including SD and simple repeat regions, together with alignment-based validation features to calculate SV genotype quality scores [44]. This approach can be applied in long-read SV callsets to enhance filtering accuracy by leveraging both read-level signals and genome context, reducing false positives in challenging regions such as SDs and tandem repeats.

Assembly-based methods require assembling reads into high-quality contigs prior to alignment. While assembly-based methods can identify larger SVs and can achieve higher consistency in SV calls, they require high coverage (>20×), and the assembly process requires significantly more computational resources. Therefore, alignment-based methods are generally preferred for most applications, as they can obtain high-quality SV callsets within a reasonable computational time and/or with limited long-read coverage.

In summary, long-read alignment-based SV callers follow similar approaches for identifying SV candidates but tend to rely on the quality of alignment evidence, which tends to limit the size and complexity of SVs, and also introduces challenges in identifying and filtering false positives for both PacBio and ONT data [34]. As each long-read SV caller employs distinct detection strategies, combining multiple tools into an ensemble is usually recommended to maximize SV detection [35]. ContextSV is a novel SV detection method that integrates evidence beyond alignments to identify structural variants that may be missed by existing tools, making it a valuable addition to SV detection pipelines.

One advantage of our method is that it incorporates long-range coverage and SNV evidence information to infer copy number and genotype directly from split alignments. We achieved this using an adapted PennCNV-Seq HMM [8, 9] to predict copy number states for each SV candidate based on coverage, SNV (single-nucleotide variant) B-allele frequency (BAF), and SNV population frequency information. While PennCNV-Seq is designed for accurately predicting copy number in specific genomic regions, our method integrates these predictions directly with read alignment evidence to improve detection performance for large SVs, relative to methods relying on read alignment evidence alone. This approach enables us to also report accurate copy number without relying on read support evidence.

Furthermore, major long-read based methods typically rely on fixed distance thresholds between breakpoints for clustering SV candidates. In regions with high alignment ambiguity and imprecise breakpoints, this approach becomes more unreliable, particularly for SVs spanning a broad range of sizes. Our method utilizes DBSCAN clustering with reciprocal overlap as the epsilon distance threshold parameter to effectively cluster breakpoint evidence for SVs of any length. This metric was originally used for comparing calls in array CGH data with imprecise breakpoints [45] and is also used by Truvari, a well-established SV benchmarking and comparison tool [13]. This approach makes ContextSV effective at clustering breakpoint evidence for large SVs, as shown by the robust identification of simulated inversions spanning several megabases (Mb) in length.

To improve precision and filter false positives, we develop ContextScore, a classification model that assigns confidence scores based on important SV validation features. The model is trained on SV caller features including read support, breakpoint read depth, and HMM likelihood to identify potential artifacts associated with false positives. The model also yields an additional score based on genomic context, including SD and simple repeat regions, as well as highly conserved regions less prone to structural variants. Additionally, missing sequences, or gaps, in the reference genome assembly are a common artifact and source of false positive SV calls. There are existing annotations for these regions, such as for GRCh38. In our tool we include support for BED files containing locations of assembly gaps, which are used to filter likely artifacts.

To highlight the advantages of our method for SV detection, we perform a benchmarking study with available datasets, including the GIAB HG002 and Platinum Pedigree whole-genome SV calling benchmark, the GIAB HG008 somatic SV calling benchmark, and simulated long-read whole-genome benchmark datasets. ContextSV consistently achieves high recall for >10 kb SVs, with variable precision. We also show a few examples of large CNVs that were identified by our method and not by others, including several experimentally validated CNVs in the KOLF2.1J cell line, and a set of simulated large clinically relevant inversions identified only by ContextSV and Sniffles2. Finally, we find that our method exhibits a high true positive rate for somatic SV detection, and we highlight a large 95 kb duplication identified only by ContextSV and Delly.

We also perform an analysis of computational performance on the HG002 ONT R10.4.1 whole genome dataset and compare with Sniffles2, cuteSV, and Delly (Supplementary Table S2). Although ContextSV shows relatively high wall clock time, CPU time, and memory, these demands stay within a reasonable range for most computing environments, making it a practical choice for large-scale SV analysis.

Taken together, these results indicate that ContextSV is particularly well-suited for use cases where maximizing sensitivity for large or complex SVs is the primary goal, such as when standard callers have failed to identify a candidate variant and a more exhaustive re-analysis is warranted. However, this sensitivity advantage comes with an elevated false positive rate, especially in the large SV size intervals where ContextSV’s recall is strongest. Users should therefore integrate ContextSV into pipelines that include downstream filtering or multi-caller validation, and should be aware that its output is best interpreted in contexts where false positives can be tolerated or subsequently resolved—for example, in diagnostic re-analysis workflows where an initial search has been uninformative and broader variant discovery is prioritized over call-level precision.

Context SV presents a few limitations. First, breakpoint detection relies on alignment evidence and is therefore limited by the read length distributions and alignment quality. This could be improved, for example by incorporating long-range optical mapping information, which could be used to provide additional supporting evidence for breakpoints in large SVs. Second, ContextScore uses a ML model trained on benchmarking datasets with a limited size and complexity of SVs, with a particularly low number of >100 kb SVs (Supplementary Fig. S5). This results in reduced classification performance for larger SVs. The limited number of benchmarking datasets may also limit generalizability of ContextScore to independent datasets with different SV distributions, sequencing coverage, and error profiles. We estimate generalizability using a per-chromosome cross-validation approach, but to estimate true generalizability to independent samples requires more rigorous hold-out validation spanning multiple benchmarks and sequencing platforms not included in the training pool, to avoid dataset-specific biases. Finally, regions of the genome with complex ploidy or mapping ambiguity, such as pseudoautosomal regions (PARs), present inherent challenges for SV calling. Reads originating from PAR1 and PAR2 may map ambiguously between chromosomes X and Y, potentially inflating false positive SV calls in these regions [46, 47]. Similarly, copy number variants and mosaic ploidy changes can confound read-depth and split-read signals used by alignment-based callers. For regions of the genome with complex ploidy or mapping ambiguity, this method does not explicitly mask or handle these separately, but these regions can be easily excluded from analysis by the user.

In the future, we aim to build upon our SV detection framework to classify complex SVs involving multiple events. ContextSV currently is limited to single-event representations, therefore tandem events (e.g. inversion followed a deletion) may appear as multiple independent SVs or are missed entirely due to the junction reads not matching expected evidence (e.g. copy number predictions) for single events. Since the current framework expects one event per locus, nested SVs with reads supporting an outer vs. inner event may also provide conflicting evidence for single SV events. Multi-breakpoint events such as translocations also do not map to any supported signature in this framework. ContextSV is therefore well-suited for sensitive identification of large, single-event SVs, particularly CNVs and inversions, with improved cost and computational efficiency over assembly-based methods.

Furthermore, as our framework operates directly on read alignments, it is immediately applicable to T2T assemblies such as CHM13, which resolve repetitive and centromeric regions that are prone to missed or false SV calls in standard draft references [48]; applying our method in this context may further improve variant detection sensitivity. Finally, we aim to incorporate support for alignment to reference pangenome graphs. Pangenome graphs can better capture diversity in human genomic variants across populations by representing alternate sequences as diverging paths relative to haplotypes contained in the graph. Alignments to the graph are analogous to linear reference genome alignments, and therefore we can leverage our approach to integrate evidence and refine SV candidates from graph alignments.

## Supplementary Material

lqag108_Supplemental_File

## Data Availability

Source code is available for ContextSV at https://github.com/WGLab/ContextSV (https://doi.org/10.5281/zenodo.21774707) and for ContextScore at https://github.com/WGLab/ContextScore (https://doi.org/10.5281/zenodo.21815943).
