# Supplementary material for: Long-read based detection of large copy number variants with potential functional significance using the ContextSV structural variant caller
Source: NAR Genom Bioinform. 2026 Sep 9;8(3):lqag108. doi: 10.1093/nargab/lqag108 (PMC13554283; doi:10.1093/nargab/lqag108)
Supplement: Supplementary file 1 [file lqag108_supplemental_file.pdf]

Supplementary Materials

SV candidate feature	Description
**SV type**	Structural variant type (DEL, DUP, INV, INS), encoded categorically.
**SV length (bp)**	Absolute SV length in base pairs.
**SV length bins**	Includes 50-500 bp, 500-5,000 bp, 5,000-50,000 bp, and >=50,000 bp.
**Normalized depthRead depth at the SV locus normalized by sample mean coverage.HMM log-likelihood**	Log-likelihood of the copy-number state sequence from the HMM.
**Cluster size per kb**	Number of supporting reads in the SV cluster, normalized by SV length (per kb).
**Call evidence type**	Alignment evidence category used for calling (CIGAR, clipped, split; encoded categorically).
**Alignment offset**	Offset (bp) between reference and read span for split-alignment evidence.
**Nearest same-type non-overlap distance**	Distance to nearest non-overlapping SV of the same type, represented as raw distance, log1p distance, and length-relative log1p distance.
**Local same-type SV density**	Counts of same-type SVs in +/-1 kb, +/-10 kb, and +/-100 kb windows around the SV center.

Genomic context feature	Description
**Simple repeat breakpoint overlap**	Breakpoint-level simpleRepeat overlap at both left and right breakpoints.
**Repeat span density**	Fraction of the SV span covered by simpleRepeat intervals (0-1).
**phastCons overlap**	Whether the SV interval overlaps phastCons100way conserved elements.
**Segmental duplication breakpoint overlap**	Breakpoint-level genomicSuperDups overlap at both left and right breakpoints.
**Distance to centromere/telomere**	Distance features to nearest centromere and telomere regions (both log1p transformed).
**Fragile-site overlap**	Whether the SV overlaps fragile-site intervals from HumCFS.

Supplementary Table 1. List of features used by the ContextScore machine learning model to assign a confidence score to structural variants from ContextSV.

Evaluation of Computational Performance

We compared the computational performance of ContextSV, Sniffles2, cuteSV, and Delly, summarizing results in Supplementary Table 2. We evaluate long-read SV calling performance for the full 209 GB HG002 ONT R10.4.1 whole genome dataset using an 8-core compute node with 32 GB memory and specifying 8 threads for SV calling. While ContextSV exhibits the highest wall clock time, CPU time, and memory usage, these resource requirements remain within a reasonable range for most computational environments, making it a feasible option for large-scale SV analysis.

Future improvements include reducing overall memory usage by smaller per-contig or slice-based caching of the reference genome, which may further improve runtime on large genomes. Streaming and buffered writing for large VCFs will also be implemented to reduce overhead. Finally, dynamic chromosome scheduling for balancing heavy and light tasks across threads can improve total runtime consistency.

	**ContextSV**	**Sniffles2**	**cuteSV**	**Delly**
**Wall clock time (H:M:S)**	2:01:49	0:11:13	0:12:41	6:28:06
**CPU time (H:M:S)**	3:22:26	0:57:32	1:46:26	6:23:17
**Max. memory usage (GB)**	16.299	1.321	3.857	2.696

Supplementary Table 2. Comparison of ContextSV CPU, wall clock time, and memory efficiency versus other major long-read SV callers. All results were generated using an 8-core compute node with 32 GB memory. The following is a list of the software versions used with default parameters: Sniffles2 v2.4, cuteSV v2.1.1, DELLY (ONT mode) v1.3.1. For long-read SV calling, we use the full 209 GB HG002 ONT R10.4.1 whole genome dataset.

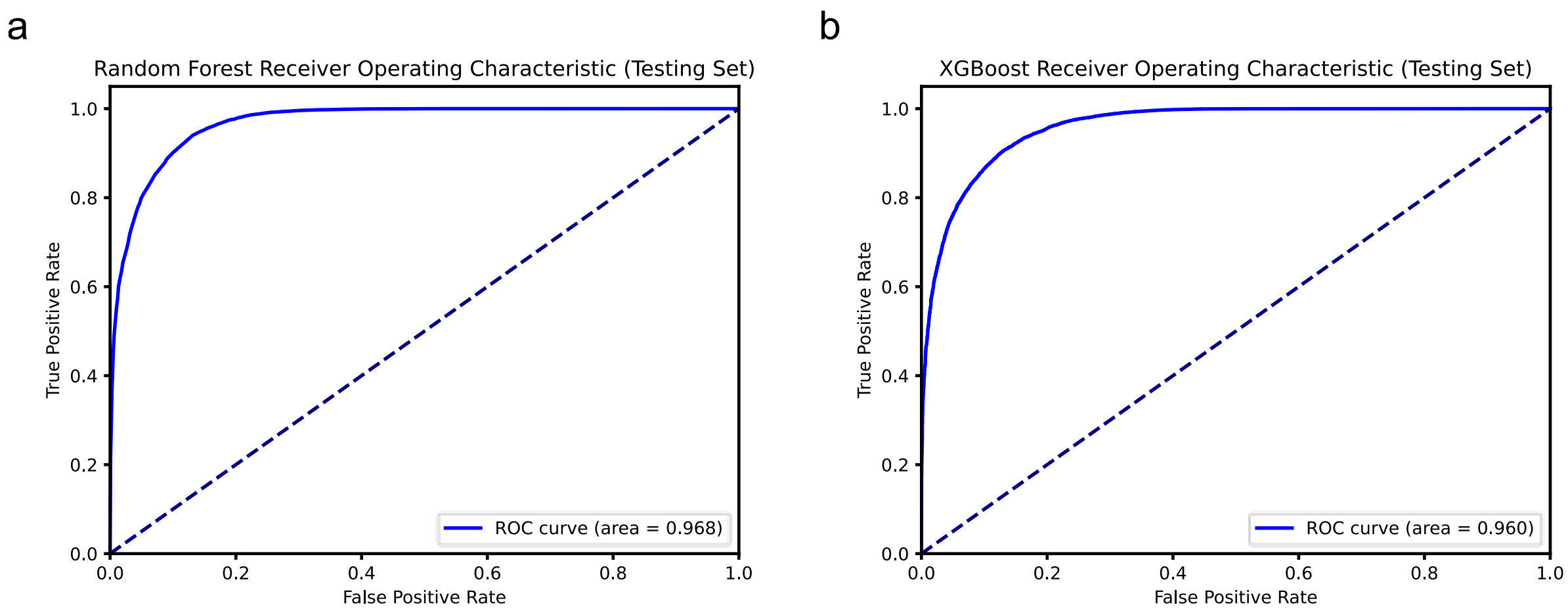

*Supplementary Figure 1. Receiver operating characteristic (ROC) curves for ContextScore evaluated on a held-out test set using an 80/20 training–testing split. Results are shown for **a,** Random Forest and **b**, XGBoost classifiers.*

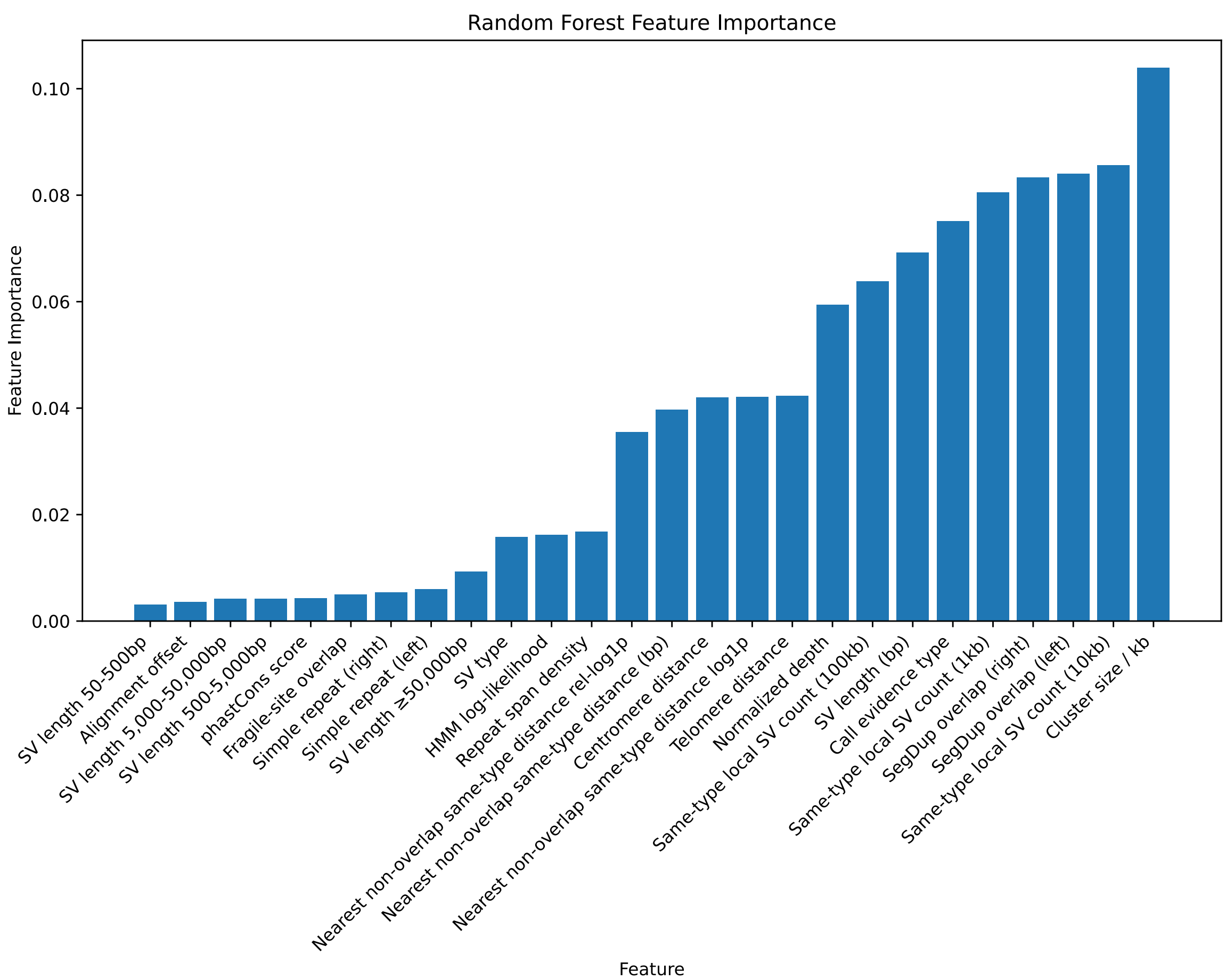

Supplementary Figure 2. Feature importances for SV call and genomic context features used in the ContextScore machine learning model for assigning SV confidence scores.

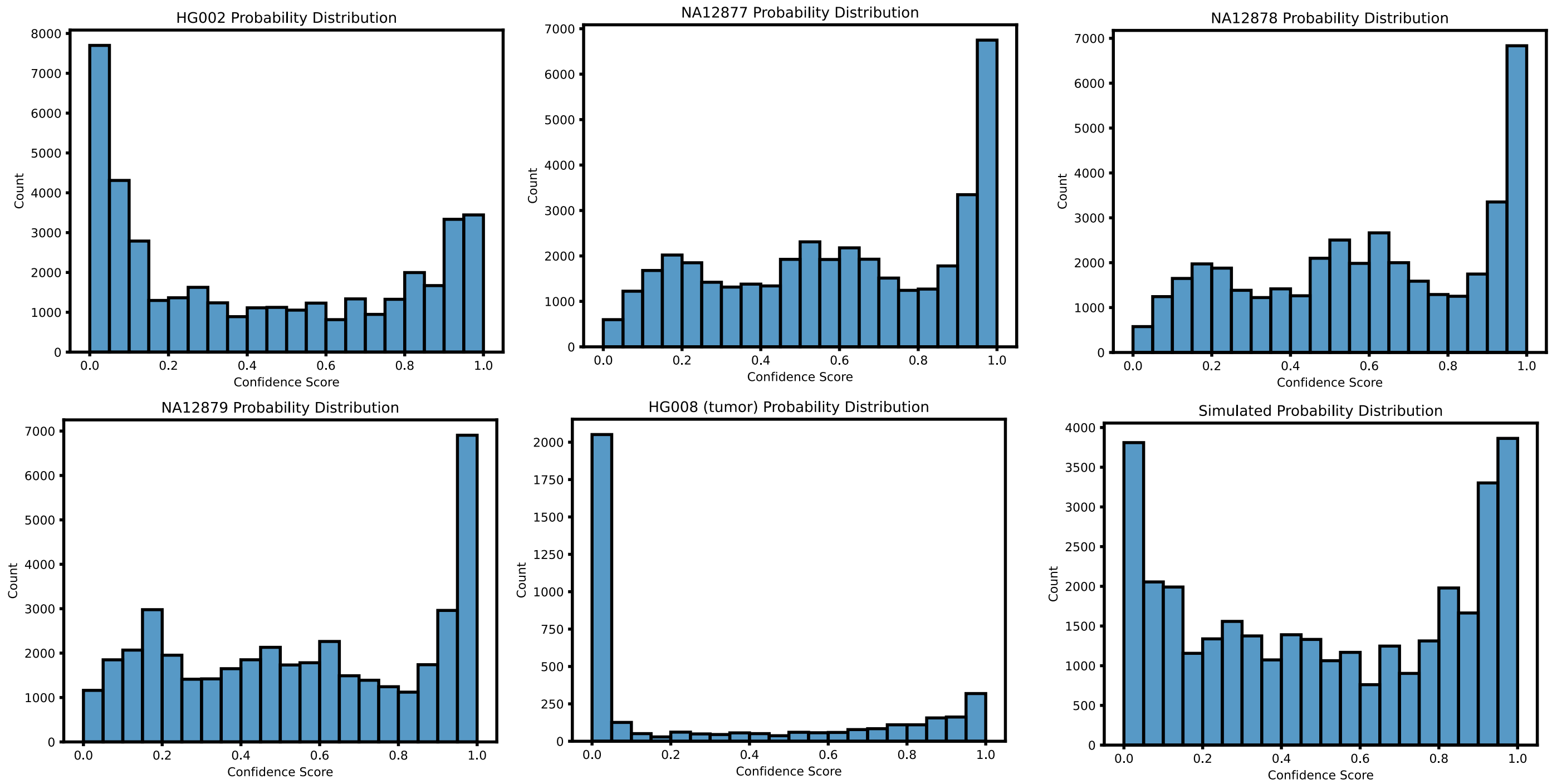

Supplementary Figure 3. ContextScore probability distributions for different samples.

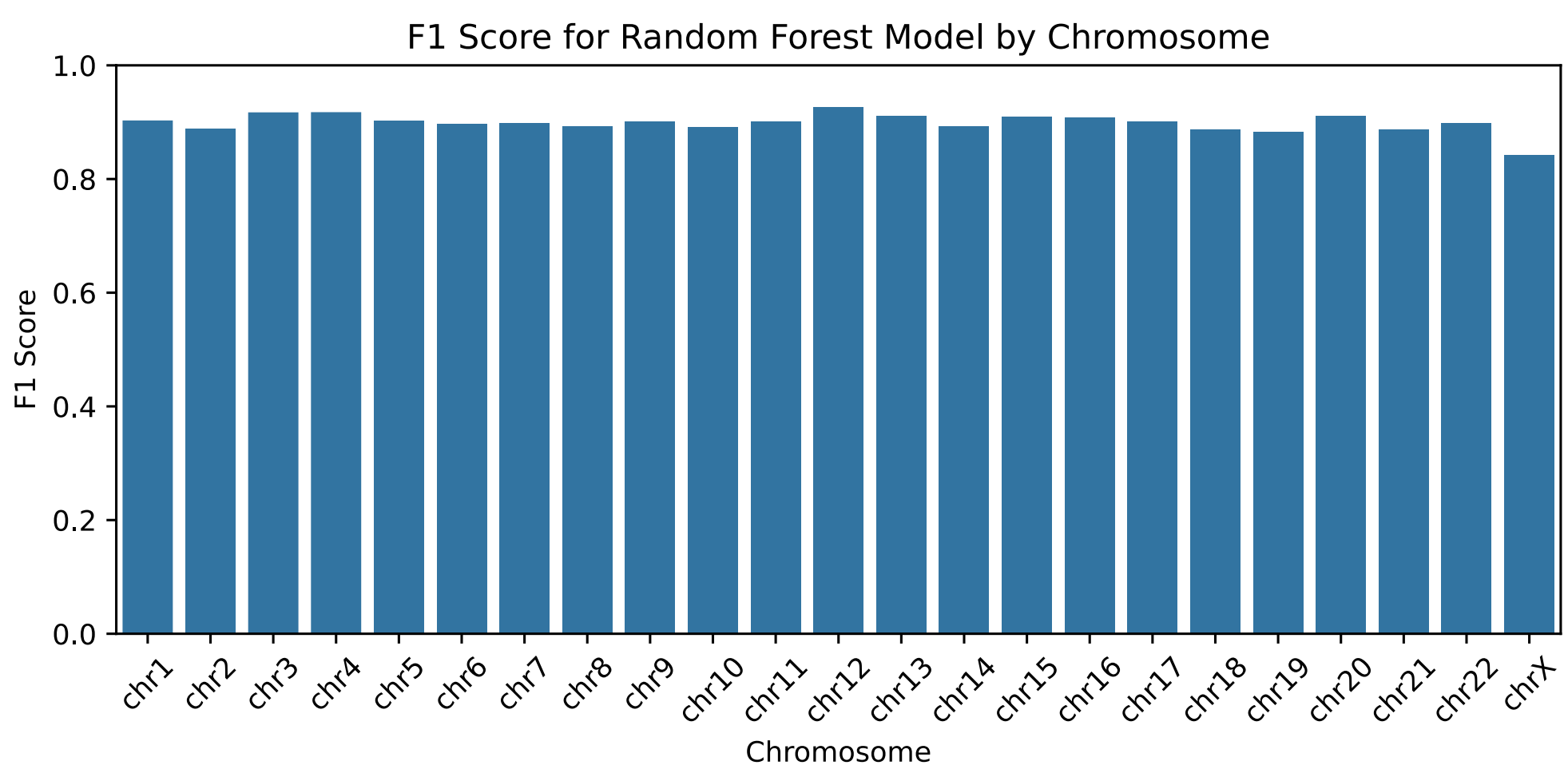

Supplementary Figure 4. F1 scores for per-chromosome cross validation of the Random Forest model.

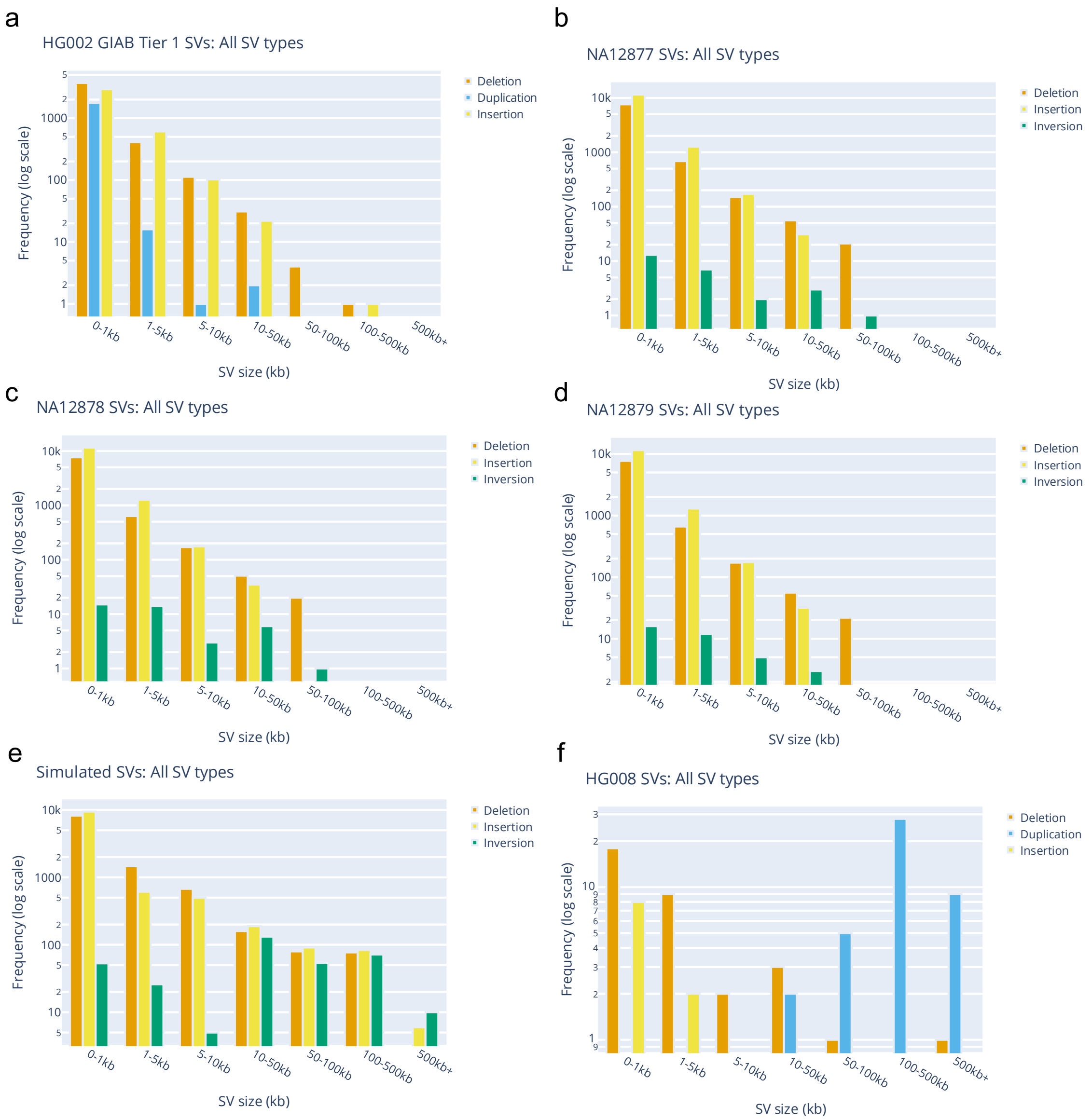

Supplementary Figure 5. SV size and type distributions for SV benchmarking datasets (with PASS filters applied). ***a***, *HG002 SVs in high-confidence regions;* ***b***, *NA12877;* ***c***, *NA12878;* ***d***, *NA12879;* ***e***, *Simulated ONT SV dataset;* ***f***, *HG008 somatic SVs.*

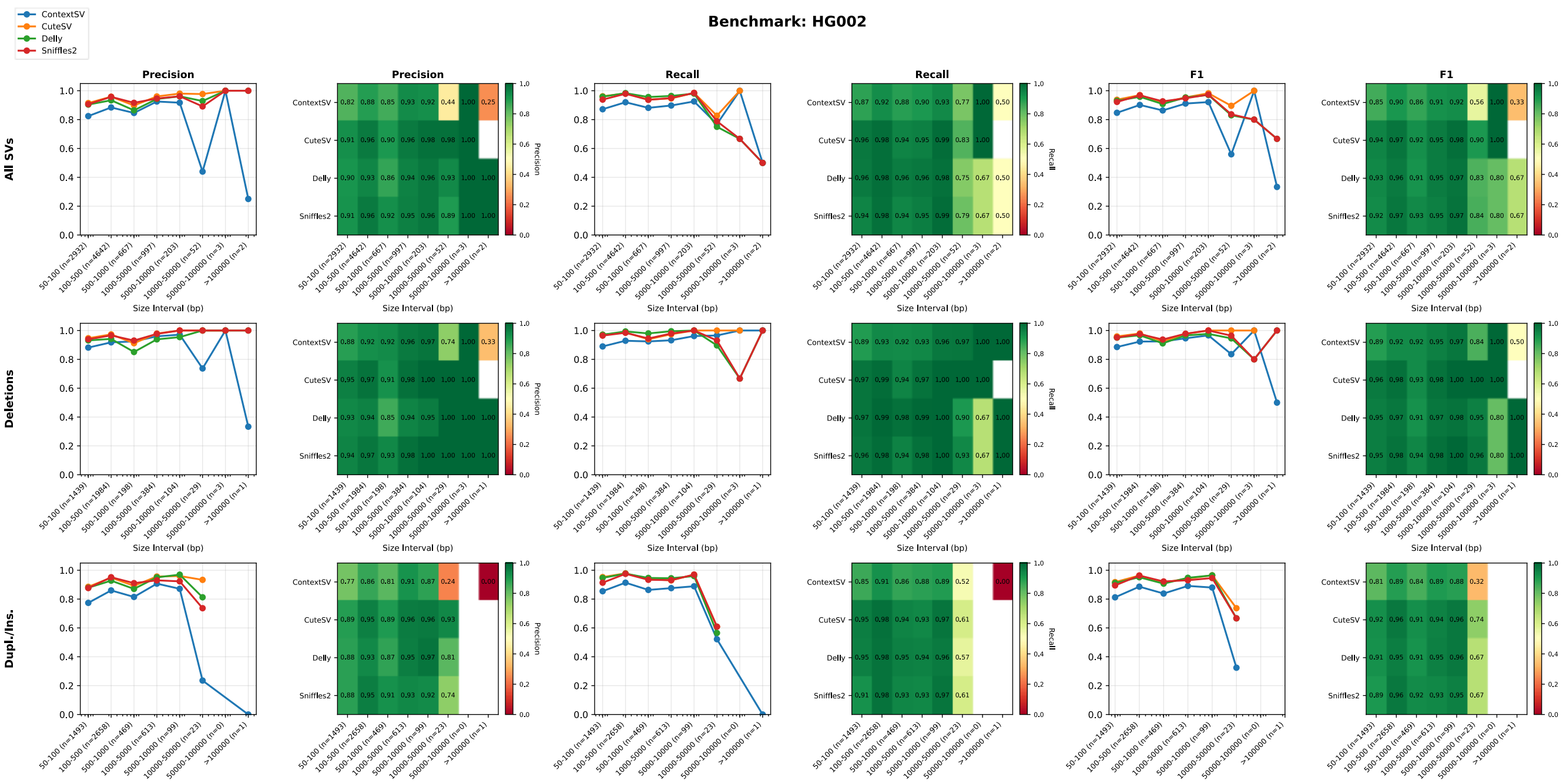

Supplementary Figure 6. Performance metrics stratified by SV type (all SV types, deletions, and combined insertions/duplications) and size interval for the HG002 benchmarking dataset.

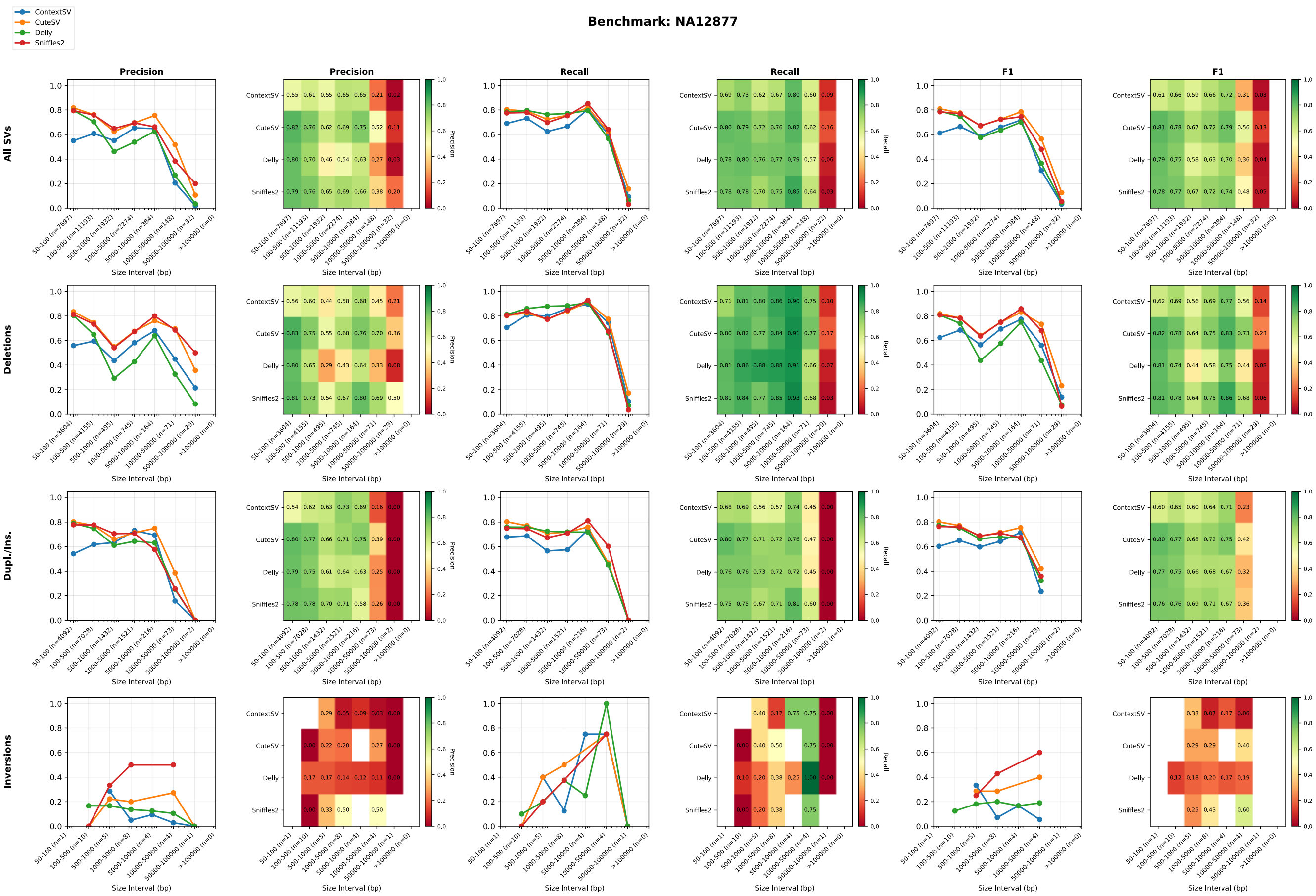

Supplementary Figure 7. Performance metrics stratified by SV type (all SV types, deletions, combined insertions/duplications, and inversions) and size interval for the NA12877 benchmarking dataset.

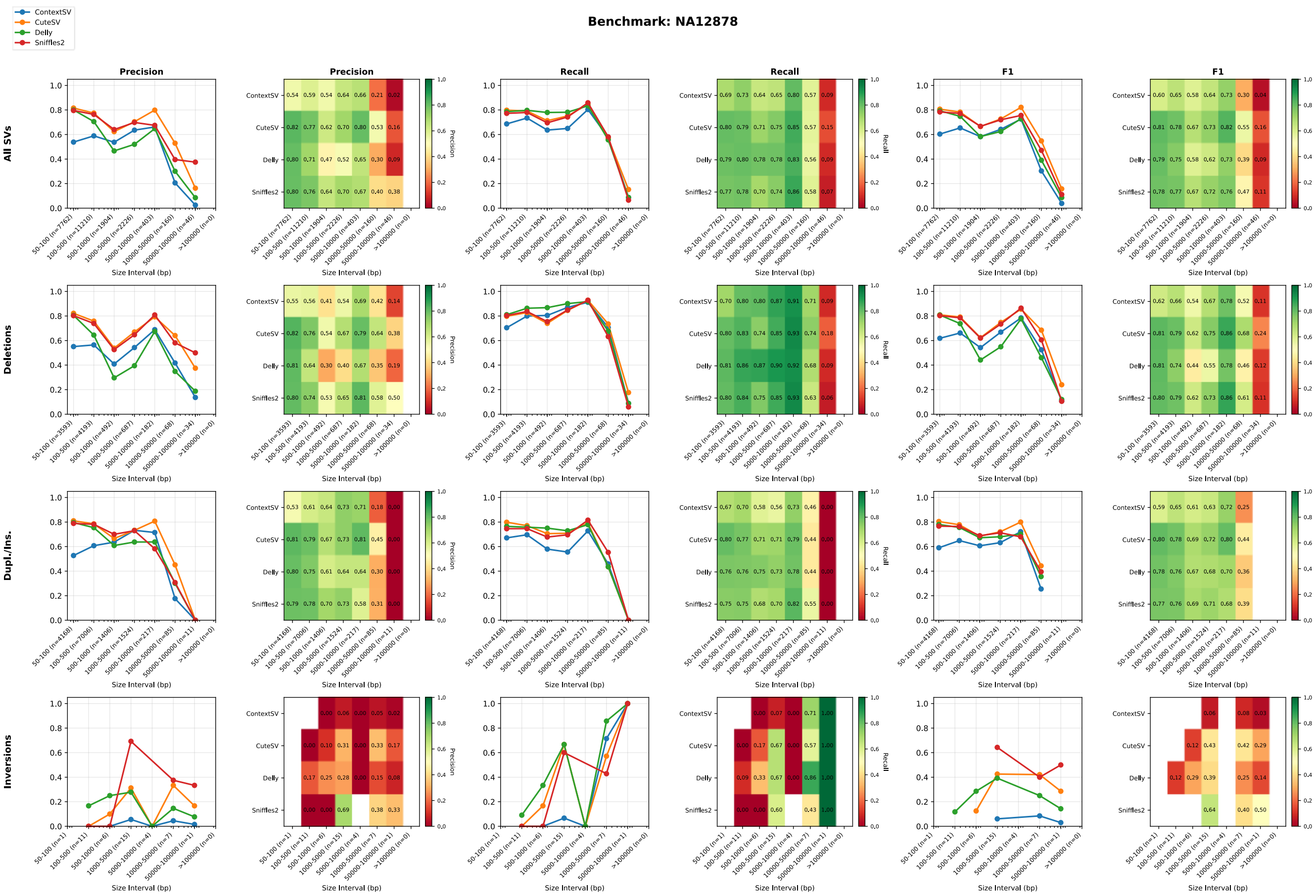

Supplementary Figure 8. Performance metrics stratified by SV type (all SV types, deletions, combined insertions/duplications, and inversions) and size interval for the NA12878 benchmarking dataset.

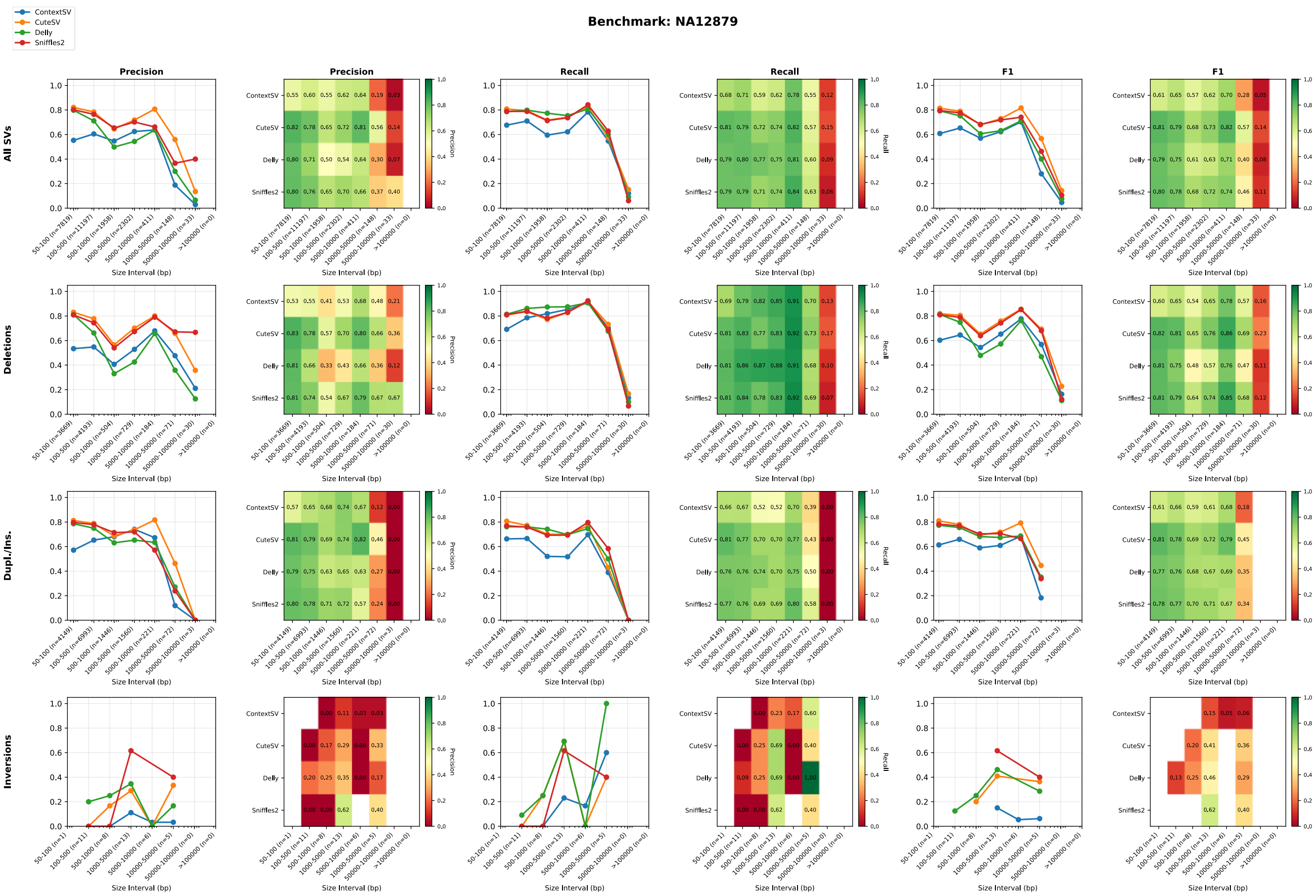

Supplementary Figure 9. Performance metrics stratified by SV type (all SV types, deletions, combined insertions/duplications, and inversions) and size interval for the NA12879 benchmarking dataset.

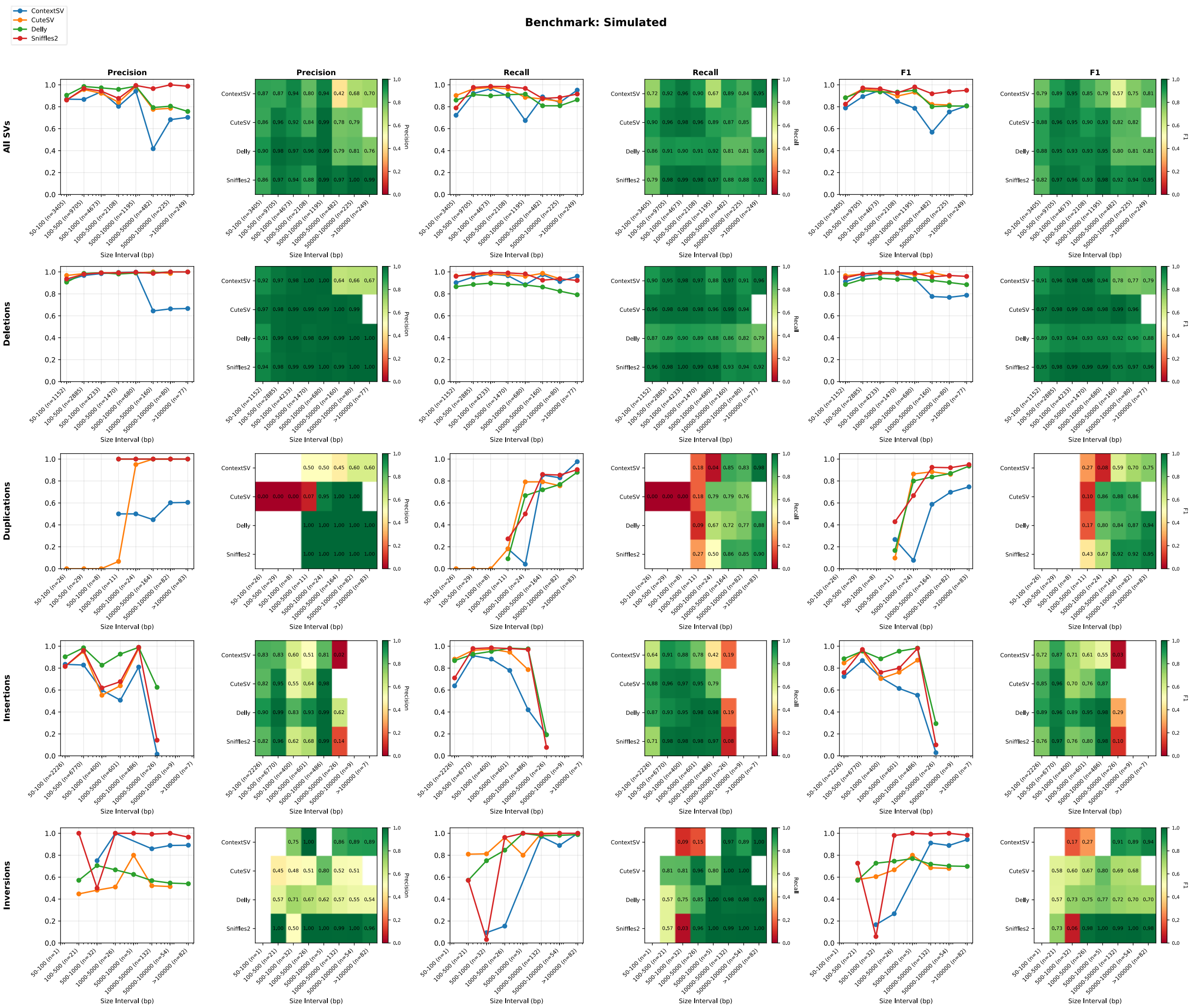

Supplementary Figure 10. Performance metrics stratified by SV type (all SV types, deletions, combined insertions/duplications, and inversions) and size interval for the simulated benchmarking dataset.
